# Advancing Air Pollution Exposure Models with Open-Vocabulary Object Detection and Semantic Segmentation of Street-View Images

**DOI:** 10.1021/acs.est.5c09687

**Published:** 2025-09-27

**Authors:** Zhendong Yuan, Jules Kerckhoffs, Pi-i Debby Lin, Esra Suel, Hao Li, Li Yi, Marcia Pescador Jimenez, Peter James, Kees de Hoogh, Gerard Hoek, Roel Vermeulen

**Affiliations:** † Institute for Risk Assessment Sciences, 8125 Utrecht University, Utrecht 3584 CM, Netherlands; ‡ Division of Chronic Disease Research Across the Lifecourse (CoRAL), Department of Population Medicine, 1811 Harvard Medical School and Harvard Pilgrim Health Care Institute, Boston, Massachusetts 02215, United States; § Centre for Advanced Spatial Analysis (CASA), 4919 University College London, London W1T4TJ, United Kingdom; ∥ Department of Geography, 37580 National University of Singapore, Singapore 119077, Singapore; ⊥ Department of Epidemiology, 27118 Boston University School of Public Health, Boston, Massachusetts 02118, United States; # Department of Environmental Health, Harvard TH Chan School of Public Health, Harvard University, Boston, Massachusetts 02115, United States; ∇ Division of Environmental and Occupational Health, Department of Public Health Sciences, Davis School of Medicine, University of California, Davis, California 95616, United States; ○ 30247 Swiss Tropical and Public Health Institute, Allschwil 4123, Switzerland; ◆ University of Basel, 4001 Basel, Switzerland; ¶ Julius Centre for Health Sciences and Primary Care, University Medical Centre, Utrecht University, Utrecht 3584CX, Netherlands

**Keywords:** mobile sensing, street-view image, vision-transformer models (ViT), vision-language model (VLM), deep learning, land use regression (LUR), exposure assessment, air pollution

## Abstract

Mobile monitoring campaigns combined with land use regression (LUR) models effectively capture fine-scale spatial variations in urban air pollution. However, traditional predictor variables often fail to capture the nuances of the built environment and undocumented emission sources. To address this, we developed a framework integrating customizable object-level and segmentation-level visual features from street-view images into stepwise regression and random-forest-based LUR models. Using 5.7 million mobile air pollution measurements (2019–2020) and 0.37 million street-view images (2008–2024), we mapped nitrogen dioxide (NO_2_), black carbon (BC), and ultrafine particles (UFP) across 46,664 road segments in Amsterdam, The Netherlands. Incorporating street-view images improved model performance, increasing *R* ^2^ by 0.01–0.05 and reducing mean absolute errors by 0.7–10.3%. Sensitivity analyses indicated that key street-view-derived visual features remained stable across years and seasons. Using images from nearby years expanded training instances, thereby enhancing alignment with mobile measurements at fine granularity. Our open-vocabulary object detection module identified influential but previously unrecognized object predictors, such as chimneys, traffic lights, and shops. Combined with segmentation-derived features (e.g., walls, roads, grass), street-view images contributed 8–18% feature importance to model predictions. These findings highlight the potential of visual data in enhancing hyperlocal air pollution mapping and exposure assessment.

## Graphical abstract

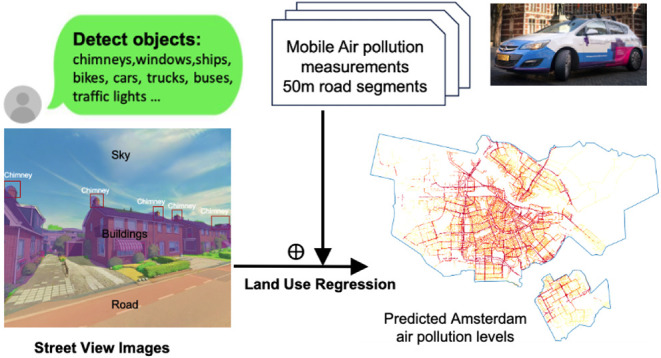

## 1 Introduction

Mobile monitoring campaigns provide fine-scale spatiotemporal air pollution data, enabling the creation of high-resolution air pollution maps. Land use regression (LUR) models, which incorporate land-use and demographic variables, are commonly used for this purpose. However, these conventional numerical predictor variables do not fully capture the complexities of the built environment and undocumented emission sources. Since street-view images are also collected via mobile platforms (i.e., cars), their level of detail closely aligns with that of mobile air pollution measurements. In the literature, street-view imagery has been broadly applied to estimate various urban environment factors such as noise^1–3^ and heat.^4–6^ Despite this, integrating street-view imagery into mobile-based air pollution modeling remains underexplored.

In the literature, one common approach to embedding street-view images in air pollution LUR models involves extracting visual features using pretrained semantic segmentation models.^7–10^ Semantic segmentation models are designed to classify each pixel into certain semantic classes (e.g., tree, sky, road, and buildings). For example, Qi and Hankey (2021)^8^ conducted a bike monitoring campaign in Blacksburg, Virginia (USA), collecting 100 h of data on black carbon (BC) and particle numbers (PN). They segmented the Google street-view (GSV) images at the pixel level into 150 classes using a pyramid scene parsing network (PSPNet) pretrained on the ADE20K data set (a segmentation benchmark data set with 20,000 manually annotated images). The resulting percentages of segmented classes (e.g., tree) formed a street-view segmentation-only model and were compared with classic LUR models, showing comparable predictive power (*R* ^2^ = 0.57–0.64 (0.50–0.57) and 0.65–0.73 (0.61–0.66) for BC and PN, respectively). More recently, a mobile monitoring study in Guangzhou, China, using low-cost sensors equipped on 314 taxis, reported that street-view segmentation-only models achieved *R* ^2^ of 0.098–0.110 for NO_2_.^11^ However, it remains unclear whether street-view features provide additional value beyond conventional LUR models, particularly when high-quality traffic intensity data are available to capture major emission sources.

Another method of processing street-view images is object detection, which counts specific objects rather than classifying pixels. Xu et al. (2022)^12^ conducted a year-long mobile monitoring campaign in Toronto, Canada (2020–2021), using cars equipped with panoramic cameras that paired air pollution measurements with street-level images. An object detection network (i.e., You only look once, YOLO) pretrained on the COCO data set (Common Objects in Context, which is an object detection benchmark data set with 200,000 labeled images) was applied to extract traffic-related features. This object-level information was then combined with segmentation-level information and meteorological information in LUR models showing adequate performance for short-term (i.e., 10-s averages) ultrafine particle (UFP) exposure predictions (*R* ^2^ = 0.66).

Computer vision models pretrained on large benchmark data sets are generally restricted by the predefined classes (defined by benchmark data sets), which impede the discovery of previously unrecognized visual features that can be used to predict air pollution concentrations. Additionally, street-view imagery poses challenges related to temporal variations. Air pollutant levels can fluctuate significantly across years and seasons,^13^ yet street-view images provide only static snapshots of the environment, typically captured during spring/summer every few years. The impact of using images that are not temporally aligned with air pollution measurements (e.g., from different years or seasons) on hyperlocal air pollution modeling requires further exploration.

Our study aims at 1) whether visual features derived from street-view images improve the accuracy of traditional LUR models and whether this improvement is influenced by the temporal attributes of the images (e.g., different years or seasons); and 2) which visual features contribute to the gain of accuracy. To address these questions, we propose a flexible and interpretable visual LUR framework (VLUR) to model hyperlocal air pollution concentrations. Two levels of visual information (i.e., object and segmentation) were extracted from street-view images. To overcome the limitations of predefined classes, we employed an open-vocabulary object detection network^14^ that enables customized object detection described in natural language and facilitates the discovery of novel diagnostic features. Air pollution measurements, collected during a mobile monitoring campaign in Amsterdam from March 2019 to February 2020, served as training inputs to estimate long-term hyperlocal concentrations of nitrogen dioxide (NO_2_), black carbon (BC), and ultrafine particles (UFP) at a 50 m road segment resolution. Sensitivity analyses were conducted using different temporal image selection strategies, including images from the specific measurement year, plus the nearest available year, and a seasonally weighted approach within LUR models.

## 2 Method

### 2.1 Amsterdam Mobile Monitoring Campaign

Our mobile monitoring campaign in Amsterdam was conducted over 10 months, from May 2019 to Feb. 2020, on weekdays (160 days), mainly between 9:00 and 20:00. Each road was measured multiple times on different days. Two Google street-view cars were equipped with 1 Hz NO_2_ (CAPS, Aerodyne Research Inc.), 1 Hz UFP (EPC 3783, TSI Inc.), and 1 Hz BC (AE33, Magee Scientific Inc.) measuring devices. We calibrated our lab-grade air quality instruments regularly by the manufacturer before the campaign and performed multiple colocation measurements at governmental regular monitoring sites during the campaign (details in Kerckhoffs et al., 2022 ^15^). The raw mobile data consisting of GPS points paired with air pollution measurements were processed as described previously.^15^ Briefly, the preprocessing included removing improbable measurement values, winsorizing low and high values to the 2.5th and 97.5th percentile and temporally correcting data using a reference site following Kerckhoffs et al., 2022 ^15^ (details in Appendix Text S1). The processed data, totaling 5.7 million measurements, were snapped to the nearest 50-m road segment (*n* = 46,664). The final mobile road-segment values were calculated as the mean of means, averaging values per drive-pass and per individual driving day.

### 2.2 Street-View Images

Street-view images were downloaded from Google to ensure a unified quality. A total of 367,597 images were obtained for Amsterdam using a 100-m street network grid (all available images started in 2008 until 2024), following the methods described by Klompmaker et al. (2024)^16^ and Yi et al. (2024 ^17^ & 2025 ^18^). Each panorama image was assigned to the nearest 50 m road segment within a 20-m buffer. In total, 31,624 road segments were assigned street-view images (68% of all road segments in Amsterdam). Some road segments had multiple street-view images taken during different months and years, and not all road segments had images for every year. Figure 1 shows examples of street-view images in the same location but in different years and seasons. We manually assessed image quality by randomly sampling two thousand images. All sampled images were of good quality. Unlike user-uploaded Mapillary images,^19^ low-quality images are typically prefiltered by Google.

**Figure 1:**
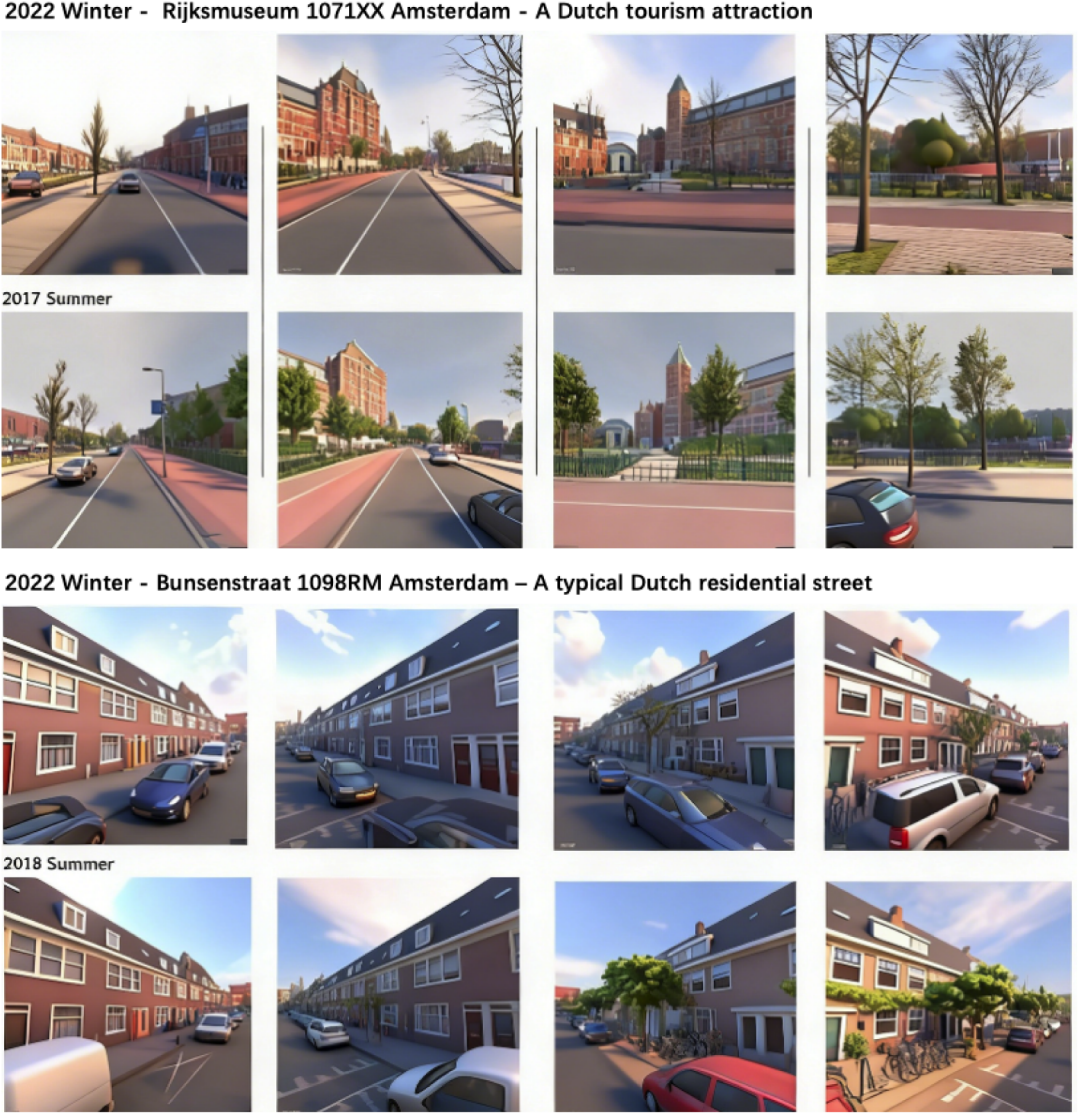
Examples of street-view images in different years and seasons. TikTok AI was used to convert street-view images into an animation style for demonstration purposes.

### 2.3 Classic LUR Model

Supervised stepwise linear regression (SLR) LUR models are often used for air pollution mapping.^20–22^ SLR selects predictor features in a forward stepwise manner to avoid collinearity. Here, the supervised stands for the addition of criteria to the stepwise selection procedure (i.e., predefined direction of effect). Details of the SLR implementation are provided in Appendix Text S2. A random forest (RF)-based LUR model was also developed to capture interaction and nonlinear relationships. The hyperparameters of the RF model, such as the number of trees, maximum depth, and maximum number of leaf nodes, were optimized using a random grid search.

The predictor variables used in the classic LUR model align with those used in our previous studies.^15,23^ These include 1) land-use data extracted from the Copernicus CORINE data set, a harmonized pan-European land-use data set;^24^ 2) traffic-related information such as traffic counts and road types derived from the Dutch national road network (NWB);^25^ and 3) population density data obtained from the Central Bureau of Statistics Netherlands (CBS).^26^ A complete list of variables is provided in Table 1, with details on buffer sizes summarized in Appendix Table S1.

**Table 1:** List of Predictor Features for Classic Land Use Regression Models

Category	Classic predictors
Land use	Agricultural land area; Airport area; Industry area; Natural and forested areas; Port area; Residential land area; Transportation area; Urban Green area; Water area
Traffic	Traffic intensity on the nearest road; Traffic intensity on nearest major road; Heavy-duty traffic intensity on the nearest road; Heavy-duty traffic intensity on nearest major road; Road length of all roads; Road length of all major roads; Traffic intensity on all roads; Traffic intensity on all major roads; Heavy-duty traffic intensity on all roads; Heavy-duty traffic intensity on major roads
Population	Population density

### 2.4 VLUR: Incorporating Visual Information in LUR

The VLUR framework incorporates an open-vocabulary object detection module and a semantic segmentation module (Figure 2). The object detection module uses OWL-ViT (a language Vision Transformer for Open-World Localization,^27^ specifically the owlvit-base-patch32 model with 153 million parameters), developed by Google and pretrained on 3.6 billion image-text pairs. Unlike commonly used object-detection models like YOLO (e.g., Xu et al., 2022 ^12^), which require manual annotation for customized classes, OWL-ViT has good zero-shot capabilities. This means that it can detect new object classes without additional training or annotations. It achieves this by replacing fixed classification layer weights with class-name embeddings (text encoders) derived from natural language, allowing it to recognize objects beyond predefined categories. This flexibility makes the use of OWL-ViT particularly effective in identifying previously unrecognized predictors. Candidate objects were selected by air pollution modeling experts, considering the potential as a possible emission source or their influence on the dispersion of air pollution. This includes trees, grass, water, ships, humans, bikes, commercial and residential buildings, gas stations, factories, construction sites, chimneys, shops, streetlamps, windows, cars, trucks, buses, traffic lights, and traffic signs. The detailed selection criteria for each object are provided in Appendix Text S3.

**Figure 2:**
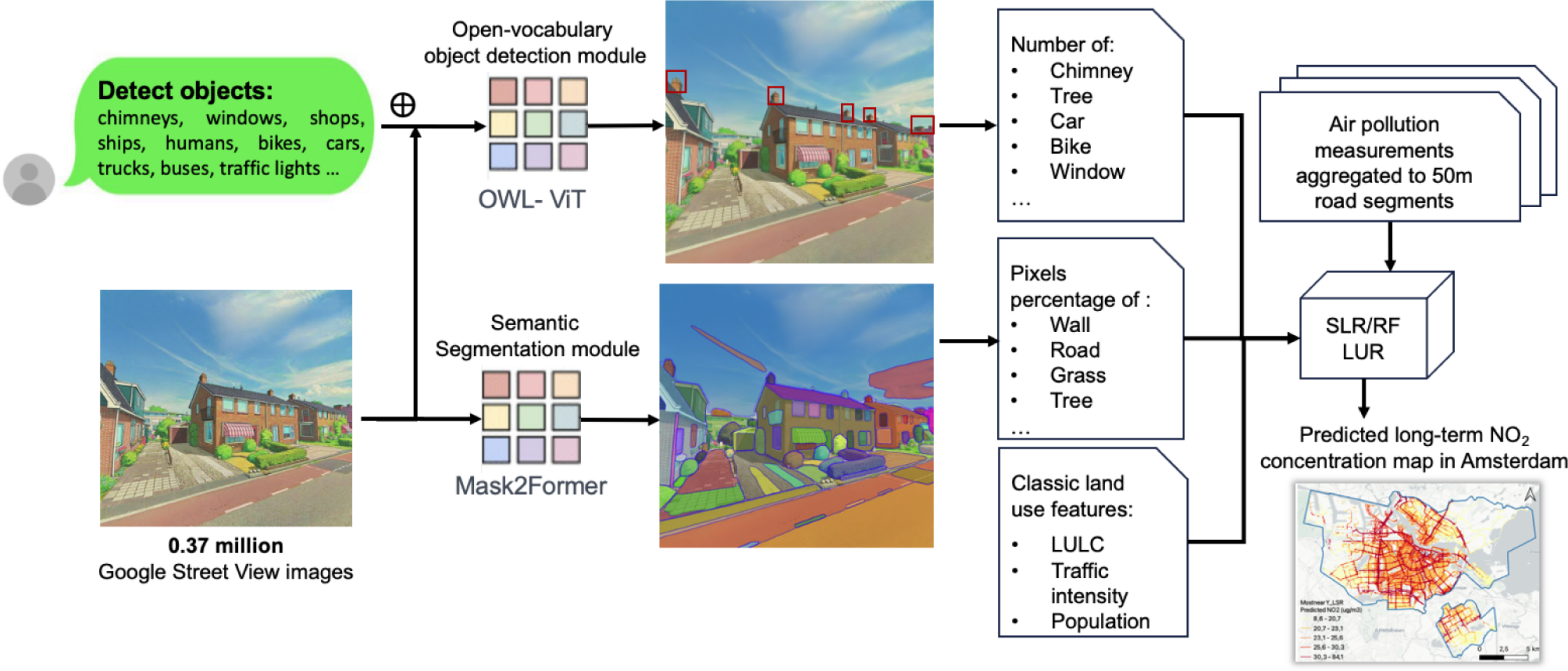
Architecture of the visual land use regression model (VLUR). Object and segmentation-level information is extracted from 0.37 million street-view images. These visual features are integrated with classic land use, traffic, and population data to train land use regression models, supervised by 50 m road-segment aggregated mobile air pollution measurements. TikTok AI was used to convert street-view images into an animation style for demonstration purposes.

The semantic segmentation module employs Mask2Former, pretrained on ADE20K with 150 predefined classes, such as wall, road, trees, grass, and sky.^28^ The combination of object detection and segmentation modules enhances VLUR’s ability to extract nuanced built environment features from street-view images. Subsequently, the object and segmentation features are combined with classic land-use features to train both linear (SLR) and nonlinear (RF) LUR models.

### 2.5 Sensitivity Test for Temporal Selection Strategy

We selected one panorama image (i.e., four directions) per road segment, based on minimal distance, taken between May 2019 and March 2020 to form the aligned specific year data set (SpecificY) and one image per road segment from the nearest available years (including the specific year) to form “the most nearby year” data set (MostnearY).

For the season-weighted model, two LUR models were developed separately for warm and cold seasons with October 31, 2019, as the dividing date. To maximize the number of available images, candidate images were chosen from the nearest available year. Corresponding air pollution measurements were averaged separately for the warm and cold seasons to align with the selected imagery. The final estimates were calculated as the mean of the two seasonal LUR model outputs. Table 2 summarizes the number of road segments for each temporal strategy.

**Table 2:** Number of Road Segments (Training Instances) under Three Temporal Selection Strategies of Street-View Images^a^

Model names		# road segwith images	# road seg with images and NO_2_	# road seg with images and BC	# road seg with images and UFP
SpecificY		19,577	19,560	19,456	19,307
MostnearY		31,624	31,604	31,406	31,094
Season-weighted	Warm	31,153	28,812	29,584	28,517
	Cold	14,476	13,694	13,124	12,647

a In total, 46,664 road segments are in Amsterdam. The large difference in the number of road segments between the warm and cold seasons is due to street-view image collection being more frequently scheduled during spring and summer.

Both SLR- and RF-based LUR models, with and without visual features, were tested across three temporal strategies. In total, 12 models (2 algorithms (SLR, RF), with and without visual features, and three temporal strategies) were developed and evaluated for each pollutant.

### 2.6 Performance Assessment and Model Interpretation

To assess the accuracy of long-term NO_2_ predictions, we used measurements from Palmes tube monitoring sites deployed by the Amsterdam Municipal Health Service (GGD) as validation data.^29^ This data set consisted of repeated 4-weekly measurements collected throughout the year, covering Amsterdam and its surroundings. We aligned this data with the time frame of our mobile monitoring campaign and selected measurements located within 20 m of the nearest road segment (*n* = 82). However, the number of validation measurements varied based on street-view image availability for each temporal strategy. Among these, 33 Palmes sites aligned with road segments across all temporal strategies.

Due to the lack of external long-term UFP and BC monitors, BC and UFP predictions were validated by 5-fold cross-validation. NO_2_ predictions in cross-validation were also reported for a more direct comparison. Squared Pearson correlation (*R* ^2^), mean absolute error (MAE), and root-mean-square error (RMSE) were used to assess the model’s performance.

SLR models were interpreted using coefficients, which indicate how much the dependent variable changes with a one-unit increase in each predictor, while keeping others constant. RF models were interpreted by Shapley values.^30^ Shapley values help interpret individual predictions by showing how each feature contributes to the difference between the actual prediction and the average prediction of the model. The street-view Shapley ratio indicates the contribution of street-view features relative to the total features.

## 3 Results and Discussion

### 3.1 Descriptive Analysis

A summary of mobile measurements across road segments for the three street-view image selection strategies is presented in Table 3. All images were processed for open-vocabulary object detection and semantic segmentation, with examples shown in Figure 3.

**Table 3:** Summary of Mobile Air Pollution Measurements across Road Segments for the Three Street-View Image Selection Strategies

Image selection strategies		NO_2_ (μg/m^3^) firstQu., median, thirdQu.	BC (μg/m^3^) firstQu., median, thirdQu.	UFP (particles/cm^3^) firstQu., median, thirdQu.
SpecificY		18.6; 23.5; 31.2	0.9; 1.2; 1.7	11,824; 17,579; 25,750
MostnearY		18.4; 23.3; 30.8	0.8; 1.1; 1.7	11,380; 17,120; 25,223
Season-weighted	Warm	17.5; 22.4; 30.8	0.8; 1.1; 1.6	11,727; 17,250; 25,790
	Cold	19.6; 26.3; 37.2	0.9; 1.3; 2.0	8,682; 15,808; 25,833

**Figure 3:**
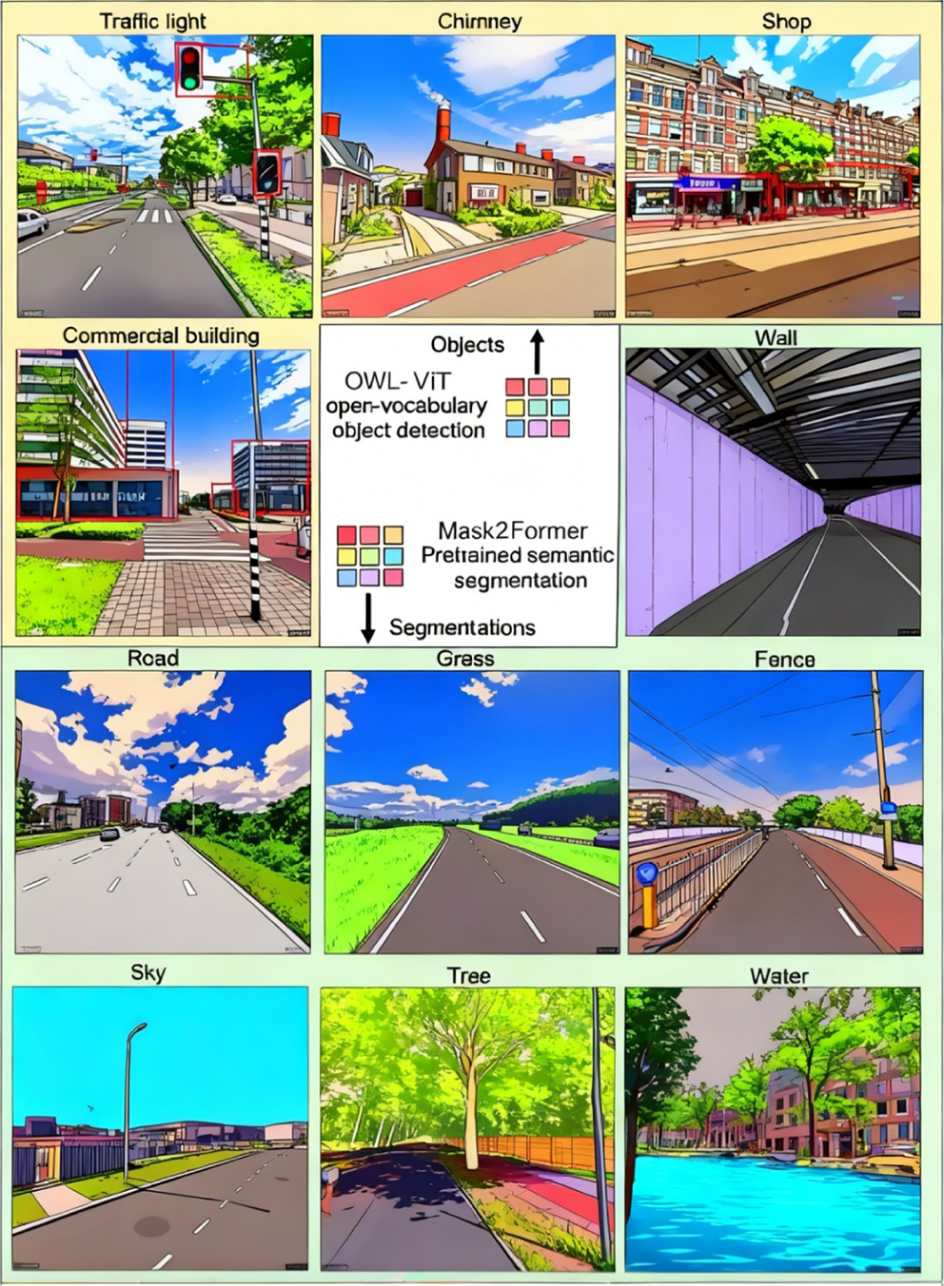
Examples of open-vocabulary object and semantic segmentation results. Objects were detected by OWL-ViT (Vision Transformer for Open-World Localization).^27^ Semantic segmentation was performed using Mask2Former.^28^ TikTok AI was used to convert street-view images into an animation style for demonstration purposes.

### 3.2 Visual Features Improve the Hyperlocal Mapping Accuracy of Air Pollution

Adding visual features improved the hyperlocal air pollution modeling for NO_2_, BC, and UFP across all temporal strategies and for both the SLR and RF models (Tables 4–6). For NO_2_, validated against independent Palmes tube measurements, incorporating visual features led to reductions of 2.1–10.3% in MAE and increases of 0.01–0.05 in *R* ^2^ values. In RF models, visual features contributed 8–11% to the importance of predictors, as estimated by Shapley values (Table 4).

**Table 4:** Performance Table of All NO_2_ Models (μg/m^3^) Trained Using Mobile Measurements and Validated by Routine Long-Term Measurements (Palmes)^a^

			All available Palmes							Palmes_33							
Temporal strategy	Algorithms	Features	# of Palmes	*R* ^2^	Change	MAE	Change	RMSE	Change	# of Palmes	*R* ^2^	Change	MAE	Change	RMSE	Change	SHAPLEY Ratio
SpecificY	SLR	Classic	43	0.47		3.85		5.15		33	0.24		4.57		5.72		
		Classic + visual		0.47	0	3.68	4.4%	4.96	3.7%		0.26	0.02	4.53	0.8%	5.55	3.0%	
	RF	classic		0.63		5.17		7.16			0.57		5.88		7.91		
		Classic + visual		**0.65**	0.02	4.89	5.5%	6.73	6.0%		0.58	0.01	5.55	5.8%	7.46	5.7%	0.09
MostnearY	SLR	classic	65	0.50		3.65		4.78		33	0.25		4.29		5.49		
		Classic + visual		0.54	0.04	**3.57**	2.1%	**4.70**	1.6%		0.27	0.02	**4.10**	4.4%	5.38	2.1%	
	RF	classic		0.57		5.66		7.69			**0.60**		5.53		7.46		
		Classic + visual		0.58	0.01	5.28	6.6%	7.29	5.1%		**0.60**	0	5.38	2.7%	7.29	2.3%	0.08
Season weighted	SLR	classic	38	0.25		4.59		5.64		33	0.23		4.40		5.68		
		Classic + visual		0.30	0.05	4.12	10.2%	5.36	5.0%		0.26	0.03	4.31	2.1%	**5.28**	7.0%	
	RF	classic		0.62		5.90		7.73			0.56		6.69		8.70		
		Classic + visual		0.61	0.01	5.70	3.5%	7.41	4.1%		0.57	0.01	6.24	6.7%	8.16	6.3%	0.10–0.11

a Validation was performed using two subsets of Palmes data, based on the availability of images. “All available Palmes” refers to Palmes data next to road segments with street-view images under specific temporal selection conditions. “Palmes_33” represents a subset of Palmes data covering all three temporal selection strategies, allowing a fair performance comparison across temporal strategies. The Shapley ratio indicates the contribution of street-view features relative to the total features.

**Table 5:** Performance Table of Black Carbon Models (μg/m^3^) Trained Using Mobile Measurements and Validated by 5-Fold Cross-Validation^a^

			5-fold Cross-validation						
Temporal strategy	algorithm	Features	*R* ^2^	Change	MAE	Change	RMSE	Change	SHAPLEY Ratio
SpecificY	SLR	Classic	0.34		0.42		0.60		
		Classic + visual	0.36	0.02	0.42	1.2%	0.60	2.8%	
	RF	classic	0.60		0.31		0.47		
		Classic + visual	0.61	0.01	0.31	1.6%	0.47	1.1%	0.12
MostnearY	SLR	classic	0.32		0.43		0.63		
		Classic + visual	0.34	0.02	0.42	2.1%	0.62	1.3%	
	RF	classic	0.59		0.31		0.50		
		Classic + visual	0.60	0.01	**0.30**	3.2%	0.49	2.0%	0.12
Season weighted	SLR	classic	0.38		0.45		0.63		
		Classic + visual	0.42	0.04	0.43	3.1%	0.59	5.1%	
	RF	classic	**0.63**		0.33		0.47		
		Classic + visual	**0.63**	0	0.33	0	**0.47**	0	0.14–0.17

a Due to the lack of long-term validation data, BC performance was validated by 5-fold cross-validation. The Shapley ratio refers to the contribution of street-view features compared to the total features.

**Table 6:** Performance Table of Ultrafine Particulate Models (particles/cm^3^) Trained Using Mobile Measurements and Validated by 5-Fold Cross-Validation^a^

			5-fold Cross validation						
Temporal strategies	Algorithms	Features	R^2^	Change	MAE	Change	RMSE	Change	SHAPLEY Ratio
SpecificY	SLR	Classic	0.11		9,533		14,679		
		Classic + visual	0.13	0.02	9,456	0.8%	14,577	0.7%	
	RF	classic	0.48		6,588		11,295		
		Classic + visual	0.50	0.02	6,372	3.3%	11,066	2.0%	0.18
MostnearY	SLR	classic	0.12		9,608		14,890		
		Classic + visual	0.14	0.02	9,536	0.8%	14,801	0.6%	
	RF	classic	0.51		6,422		11,107		
		Classic + visual	**0.53**	0.02	6,188	3.6%	10,867	2.2%	0.15
Season weighted	SLR	classic	0.22		7,938		11,249		
		Classic + visual	0.22	0	7,872	0.8%	11,178	0.6%	
	RF	classic	0.49		6,178		9,204		
		Classic + visual	0.49	0	**6,135**	0.7%	**9,143**	0.7%	0.15–0.18

a Due to the lack of long-term validation data, UFP performance was validated by 5-fold cross-validation. The Shapley ratio refers to the contribution of street-view features compared to the total features.

Within each temporal street-view selection strategy for NO_2_, SLR generally achieved a lower MAE than RF, while RF consistently produced higher *R* ^2^ values than SLR. This is mainly due to *R* ^2^ being more sensitive to variance. As shown in the density plots (Figure 4), SLR predictions closely matched the distribution of the reference Palmes data, confining the results to a narrower concentration range. In contrast, RF predictions captured a broader distribution, aligning more closely with the variability of short-term mobile NO_2_ measurements but deviating from the distribution of long-term Palmes measurements. As a result, RF achieved a higher *R* ^2^, while SLR achieved lower MAE and RMSE—a pattern observed in previous studies.^23,31^ This was previously identified as the issue of domain shift, where the training and validation data were distributed differently.^23,31^ Note that although SLR and RF models were trained with the same mobile measurements, the inherently simpler structure of SLR led to predictions that more closely aligned with the distribution of the longer-term Palmes measurements. To ensure fair comparisons across temporal selection strategies, model predictions were validated against the same subset of Palmes tubes (*n* = 33, Table 4). The MostnearY strategy with SLR achieved the lowest MAE (4.10 μg/m^3^), while the same strategy with the RF model yielded the highest *R* ^2^ (0.60). Additionally, the season-weighted strategy with SLR achieved the lowest RMSE (5.28 μg/m^3^).

**Figure 4:**
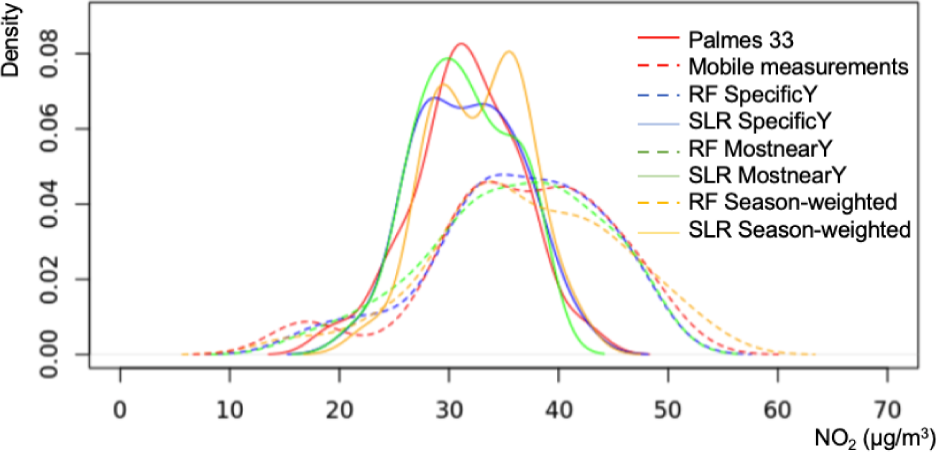
Density plot of mobile training data, model predictions, and long-term validation data for NO_2_ at 33 Palmes locations. RF: random-forest-based LUR model. SLR: stepwise linear regression-based LUR model. SpecificY, MostnearY, and Season-weighted represent temporal strategies based on different sets of street-view images. Palmes 33 is a subset of long-term fixed-site measurements of NO_2_ in Amsterdam. The LUR models were trained by using mobile NO_2_ measurements and aimed to estimate long-term NO_2_ distributions represented by Palmes data.

For BC, Table 5 shows that incorporating street-view visual features improves the cross-validation performance across all temporal image selection strategies. Models with visual features modestly outperform those without with *R* ^2^ improvements of 0.01–0.04 and MAE reductions of up to 3.1%. Similarly, for UFP, models with visual features exhibit cross-validation performance improvements of 0–0.02 in *R* ^2^ and reductions of 0.7–3.6% in MAE, compared to models without visual features (Table 6). These findings align with those of previous studies. For example, Lloyd et al. (2023)^32^ reported that combining satellite imagery with land-use features during year-long mobile monitoring campaigns in Montreal and Toronto, Canada, improved cross-validated *R* ^2^ by 0.01–0.02 for UFP and BC compared to models using only land-use features. In an earlier study, Lloyd et al. (2021)^33^ found that incorporating both street-view and satellite imagery into LUR models during a 20-day mobile monitoring campaign in Bucaramanga, Colombia, improved cross-validated *R* ^2^ by 0.06–0.07 compared to classic LUR models (UFP: 0.54 vs 0.47; BC: 0.51 vs 0.45).

For the temporal strategies, the MostnearY strategy in RF achieved the lowest MAE (0.30 μg/m^3^) for BC, while the season-weighted RF model recorded the highest *R* ^2^ (0.63) and lowest RMSE (0.47 μg/m^3^). For UFP, the MostnearY strategy achieved the highest cross-validated *R* ^2^ of 0.53, whereas the season-weighted RF model achieved the lowest MAE (6135 particles/cm^3^) and RMSE (9143 particles/cm^3^) (Tables 5 and 6). Two of the three accuracy metrics indicate that the season-weighted strategy is slightly more suitable for modeling BC and UFP.

For all three pollutants (NO_2_, BC, and UFP), the MostnearY and season-weighted strategies performed similarly, and both were better than the SpecificY strategy (Tables 4–6). Including images from different years increased the number of training instances, leading to higher accuracy. This suggests that leveraging images from multiple years provides more benefit than the drawback of introducing variations from different collection periods.

As shown in Figure 1, the most noticeable visible change between the 2018 winter and the 2022 summer was the intensity of greenery, whereas other key influential features (Section 3.3), such as the number of chimneys and the percentage of roads and walls, remained largely unchanged. This indicates that for air pollution modeling street-view-derived visual features are relatively stable across years and seasons. This also explains why the MostnearY and Season-weighted strategies produced similar overall performance.

Notably, the season-weighted strategy frequently achieved the lowest RMSE across pollutants and temporal image selection strategies. Since RMSE places greater emphasis on larger errors, this suggests that the season-weighted strategy effectively reduces extreme prediction errors, in part due to the averaging effect of the two seasonal models.

### 3.3 Model Interpretation Discovered Novel and Influential Visual Features

Several visual features were identified as relevant for mapping hyperlocal air pollution concentrations. All SLR models’ coefficient tables (including direction, slope, and significance) are in Appendix Tables S2–S19, with simplified model examples provided in eq 1. Table 7 summarizes the visual features selected by the SLR models for the strategies of SpecificY and MostnearY. In contrast, RF models did not rank any visual features among their top 20 most influential predictors, as traffic intensity features dominated (Appendix Figures S1–S3). However, Shapley values in the RF models revealed that street-view features contributed 8–11% to the predictive power for NO_2_, while their contributions were higher for BC (12–17%) and UFP (15–18%), as shown in Tables 4–6.

$${\mathrm{MostnearY NO}}_{2}\mathrm{estimations}=\mathrm{Intercept}+0.17\times \mathrm{traffic.light}+35.6\times \mathrm{wall.perc\_pixels}+11.6\times \mathrm{road.perc\_pixels}+11.2\times \mathrm{grass.perc\_pixels}+\beta_{1}\times LU+\beta_{2}\times TI+\beta_{3}\times Pop$$

$$\mathrm{MostnearY}\;BC\;\mathrm{estimations}=\mathrm{Intercept}+0.013\times \mathrm{traffic.light}+2.3\times \mathrm{wall.perc\_pixels}+0.6\times \mathrm{road.perc\_pixels}+0.7\times \mathrm{grass.perc\_pixels}+0.2\times \mathrm{sky.perc\_pixels}+\beta_{1}\times LU+\beta_{2}\times TI+\beta_{3}\times Pop$$

$$\mathrm{MostnearY}\;UFP\;\mathrm{estimations}=\mathrm{Intercept}+561\times \mathrm{chimney}+17670\times \mathrm{wall.perc\_pixels}+15430\times \mathrm{road.perc\_pixels}+15570\times \mathrm{grass.perc\_pixels}+4772\times \mathrm{tree}+\beta_{1}\times LU+\beta_{2}\times TI+\beta_{3}\times Pop$$

**Table 7:** Visual Features Selected by SLR

Pollutant	Period	Level of features	Selected Features (MostnearY)	Selected Features (SpecificY)
NO_2_	Year	Object Segmentation	Traffic light Road, wall, grass	Traffic light Road, wall, grass, fence
BC	Year	Object Segmentation	Traffic light, shop Road, wall, grass, tree, sky	Traffic light, chimney, Road, wall, grass, fence
UFP	Year	Object Segmentation	Chimney Road, wall, grass, tree	Chimney Road, grass, tree

Equation 1. Examples of SLR models with street-view features. The complete coefficient tables are in Tables S2–S19.

Segmentation features provide broad descriptions of the built environment, such as walls, roads, grass, and other general categories, all of which are positively associated with air pollution levels (Appendix Tables S2–S19). For example, a higher proportion of road pixels often indicates wider or major roads, while wall pixels often correspond to the wall of tunnels (Figure 3), commonly associated with busy roads or highways. Grass, a component of greenness, was counterintuitively associated with higher air pollution levels. In street-view images, grass often appears in highway medians or roadside strips, indirectly signifying proximity to major roads. Note that total urban greenery may not be well captured by street-view images, as the pictures are all taken from roads and, therefore, miss parks and other off-road green areas. Future research could integrate satellite images for a more comprehensive assessment.

In addition to common segmentation features such as road, wall, and grass, SLR models also selected fences (directions and slopes in Tables S1–S18). UFP models selected trees, while BC models showed a mixed pattern of selecting fences, trees, and sky. These features were all positively associated with air pollution levels. Fences were linked to highways (e.g., tunnel), railways, or industrial buildings, reflecting heavy traffic or industrial activity (Figure 3). Sky pixels, representing the openness of a location, were expected to indicate better air dispersion. However, images with a high proportion of sky often depicted industrial areas (e.g., low warehouses) or open highways without surrounding buildings. Trees, which can create urban canyons, may trap particles such as UFP and BC, leading to localized particle accumulation.^34^

Object features provide nuanced descriptions of the built environment such as chimneys, traffic lights, and shops. Traffic lights, often located on busy roads, are frequently selected by NO_2_ LUR models. Vehicles idling or accelerating at traffic lights emit higher levels of pollutants, contributing to localized accumulation that traffic intensity metrics alone cannot fully capture.

The inclusion of chimneys as predictors in the UFP and BC models highlights the diverse emission sources of these pollutants. UFP and BC are previously found to be strongly linked to vehicle and aircraft exhaust, as reflected in LUR models using traffic and airport variables.^35–37^ However, residential combustion, such as cooking, heating, and wood-burning, also has been reported as significant emissions of UFP and BC.^38^ For example, residential biomass burning accounted for 38% of ambient BC in Helsinki, Finland, while traffic emissions contributed 57%.^39^ Wood-burning stoves, pellet stoves, and fireplaces likely release particles via chimneys that can serve as proxies for such activities. However, current LUR models often do not include emissions from residential combustion, primarily due to the lack of registries of such sources. Our VLUR model addresses this gap by integrating these undocumented emission sources into the LUR framework and demonstrating a positive association between chimney density and outdoor UFP and BC levels. Since we cannot determine whether these chimneys were actively in use at the time of air pollution measurement, particularly as most mobile monitoring drives occurred during working hours on weekdays, the estimated feature importance of chimneys is likely highly underestimated. Nevertheless, their influence on UFP and BC concentrations remains strong enough to be detected as a significant emission source.

In the study in Toronto, truck numbers were positively associated with short-term UFP levels.^12^ Although our object detection was configured to identify vehicles, such as cars, trucks, and buses, none were selected as key predictors in our models. One possible reason is that street-view images are not temporally paired with mobile measurements. Unlike the real-time paired images and measurements used in Toronto,^12^ Google street-view images provide only static snapshots of road conditions. These images include both moving and parked vehicles, making it more difficult to accurately estimate traffic volumes at the precise moment of measurement. Additionally, our models incorporated traffic intensity features, which provide a more stable and precise representation of traffic patterns over time. This likely reduced the impact of vehicle-specific street-view-derived features in predicting air pollution levels.

### 3.4 Strengths and Limitations

Aggregating air pollution measurements and street-view images to 46,664 road segments, we evaluated the contribution of street-view visual information to hyperlocal mapping of BC, UFP, and NO_2_ at the city scale. Unlike prior studies that relied on shorter campaigns (e.g., 120-h^8^ or 20-day^33^ focused on BC and UFP), and were heavily dependent on semantic segmentation, our VLUR framework integrates both object-level and segmentation-level visual features. The result demonstrated a consistent improvement in the accuracy of visual land use regression (VLUR) models compared to traditional LUR models, with increases in *R* ^2^ ranging from 0.01 to 0.05 and reductions in mean absolute error between 0.7% and 10.3% under the presence of already strong predictor variables such as traffic intensity features.

Moreover, different from end-to-end approaches that directly predict pollution from images (e.g., Lloyd et al., 2021 and 2023),^32,33^ our VLUR approach offers greater interpretability while maintaining comparable performance improvements. For instance, we identified previously unrecognized object predictors and found that street-view-derived visual features contributed 8–18% to the overall feature importance in model predictions for hyperlocal air pollution levels. Our proposed VLUR model incorporates chimneys as predictive features, representing a previously neglected microemission source - the residential combustion. However, further investigation is needed to establish causality between the built environment and air pollution levels.

Our customizable open-vocabulary object detection module enabled the identification of previously unrecognized visual predictors. This is a huge step forward as it overcomes the constraints of the predefined segmentation classes, enabling more targeted feature engineering. Beyond air pollution modeling, this method has potential applications for other urban environmental factors such as noise pollution and heat exposures. Because users can define tailored visual objects in natural language, this approach allows flexible adaptation to various research needs. This approach contributes to a broader discovery-driven methodology for identifying environmental determinants of human health, supporting urban exposome research.^40^ Future air pollution research could refine the selection of object candidates to capture finer spatial variations in traffic flow, such as the detection of speed limit signs, which may correlate with distinct emission profiles. Nevertheless, the temporally sparse nature of GSV imagery inherently remains limited in capturing the nuanced temporal dynamics of traffic flow. A promising direction is to enhance the VLUR model with high-quality traffic dynamic data and fine-temporal fixed-site measurements to produce air pollution maps at fine spatiotemporal resolutions.

Although we tested linear and nonlinear LUR models under three temporal strategies, the performance improvement of VLUR over classic LUR models was only moderate. However, this was achieved with already strong and robust predictor variables, particularly traffic intensity features (strong correlations with visual features are shown in Table S20), which already explain much of the air pollution variations, as traffic is a major emission source for NO_2_, BC, and UFP. This reduced the added explanatory power of street-view features. However, high-quality traffic intensity data is not always available across all regions.^8,12,32,33^ When we removed the traffic intensity features, the *R* ^2^ increased from 0 to 0.02 to 0–0.09 for NO_2_ in cross-validation. Similarly, in the external validation using Palmes fixed-site measurements, the *R* ^2^ improved from 0 to 0.05 to 0.02–0.08 for NO_2_. The feature contribution ratio of visual features also increased from 12–17% to 18–23% (Appendix Tables S3 and S4). These findings suggest that visual features play a more prominent role when traffic intensity features are unavailable.

We downloaded street-view images using a 100m grid, resulting in 68% of 50 m road segments containing street-view images. A denser grid could improve completeness but would significantly increase data volume and financial cost. For locations that are away from roads and those without street-view images, nonvisual models may complement street-view-based models for a more comprehensive spatial coverage.

Another limitation of this study is the lack of external long-term validation for BC and UFP, a challenge commonly faced in air pollution research. Similar studies in Canada and the US have also lacked such data.^23,31,41^ While cross-validation is a widely accepted method, it does not fully address domain issues caused by the differences in distribution between long-term and mobile measurements, which may affect the downstream epidemiological applications.^23,41^

Our interpretable and customizable VLUR framework demonstrates that incorporating street-view images enhances the performance of classic land-use regression models for mapping city-scale hyperlocal air pollution concentrations. Sensitivity analyses on temporal image selection strategies indicate that for air pollution modeling, the key influential street-view-derived visual features remain relatively stable across years and seasons. The model incorporating images from multiple years around the air pollution measuring period performed comparably to the seasonally stratified model, with both yielding higher accuracy than models relying solely on images aligned with the measuring year. Additionally, integrating the customizable object detection module uncovered previously unrecognized predictors, such as the number of chimneys, which served as a proxy for undocumented emission sources and was found positively associated with BC and UFP levels. The proposed open-vocabulary object detection model facilitates the discovery of novel visual features, not only for air pollution modeling but also for various other urban environmental models. Given that imagery data is becoming increasingly omnipresent through the expansion of street-view, satellite, and crowdsourced visual data sets, VLUR is well-positioned to scale for large-scale urban environmental exposure assessments.

## Supplementary Material



## Notes

The appendix includes text describing the preprocessing of mobile measurements, the developing steps of the SLR model, tables summarizing the predictor variables and the trained SLR models, as well as figures of Shapley values of the trained RF models (PDF)
