# Supplementary material for: Advancing Air Pollution Exposure Models with Open-Vocabulary Object Detection and Semantic Segmentation of Street-View Images
Source: Environ Sci Technol. 2025 Sep 27;59(39):21237–47. doi: 10.1021/acs.est.5c09687 (PMC12509304; doi:10.1021/acs.est.5c09687)
Supplement: Supplementary file 1 [file es5c09687_si_001.pdf]

Supplementary Information for:

Advancing Air Pollution Exposure Models with Open-Vocabulary Object Detection and Semantic Segmentation of Street View Images

Zhendong Yuan[1*], Jules Kerckhoffs[1], Pi-i Debby Lin[2], Esra Suel[3], Hao Li[4], Li Yi[2], Marcia Pescador Jimenez[5], Peter James[6,7], Kees de Hoogh[8,9], Gerard Hoek[1], Roel Vermeulen[1,10]

[1] Institute for Risk Assessment Sciences, Utrecht University, 3584CM Utrecht, Netherlands

[2] Division of Chronic Disease Research Across the Lifecourse (CoRAL), Department of Population Medicine, Harvard Medical School and Harvard Pilgrim Health Care Institute, Boston, Massachusetts 02215, United States

[3] Centre for Advanced Spatial Analysis (CASA), University College London, London W1T4TJ, United Kingdom

[4] Department of Geography, National University of Singapore, 119077, Singapore

[5] Department of Epidemiology, Boston University School of Public Health, Boston, Massachusetts 02118, United States

[6] Department of Environmental Health, Harvard TH Chan School of Public Health, Harvard University, Boston, Massachusetts 02115, United States

[7] Division of Environmental and Occupational Health, Department of Public Health Sciences, University of California, Davis School of Medicine, Davis, California 95616, United States

[8] Swiss Tropical and Public Health Institute, 4002 Basel, Switzerland

[9] University of Basel, 4001 Basel, Switzerland

[10] Julius Centre for Health Sciences and Primary Care, University Medical Centre, Utrecht University, 3584CX Utrecht, Netherlands

*Corresponding author: *z.yuan@uu.nl*

Text:

Text S1. Preprocessing of mobile measurements.

Text S2. Model implementation of stepwise linear regression.

Text S3. Specific criteria for each selected street view object.

Tables:

Table S1. Spatial predictor variables with units, predefined directions of effect, and buffer sizes in Amsterdam.

Table S2. Performance table of NO_2 models (μg/m^3) trained using mobile measurements and validated by 5-fold cross-validation.

Table S3. Performance table of NO_2 models without traffic intensity features (μg/m^3) trained using mobile measurements and validated by 5-fold cross-validation.

Table S4. Performance of NO_2 models without traffic intensity features (μg/m^3) validated by

routine long-term measurements (Palmes).

Table S2-S19. Coefficients of stepwise linear regression under three image selection strategies (i.e., SpecificY, MostnearY, and Season-weighted) for three pollutants (NO_2, BC, and UFP).

Table S20. Correlation table of traffic intensity features against selected visual features.

Figures:

Figure S1. Global Shapley summary plot of RF for NO_2.

Figure S2. Global Shapley summary plot of RF for BC.

Figure S3. Global Shapley summary plot of RF for UFP.

Text S1. Preprocessing of mobile measurements

Due to malfunctioning and internal calibration of the instrument, measured 1-second NO2 values higher than 500 μg/m^3 and lower than 0 were discarded (0.9 % of measurements). For BC, we removed values higher than 30 μg/m^3 (0.1 % of the measurements), and for UFP, we removed values higher than 500,000 (0.1 % of the measurements) particles/cm^3 from the data as such values are not physically possible in the measured environments.

For the temporal correction, a difference correction method was used for Amsterdam, using background monitoring stations from the Dutch National Air Quality Monitoring Program. First, an overall mean concentration of the whole measurement campaign was calculated at the reference station. Then, a moving average (4 hours) was calculated at the same reference station. Finally, for each (1-Hz) measurement of the street-view cars, the 4-hour moving average was subtracted from the overall mean concentration and subsequently subtracted or added to the measured data.

Text S2. Model implementation of stepwise linear regression

The stepwise linear regression (SLR) model selects predictor variables in a forward stepwise manner. It starts by taking an empty, intercept-only model, and then adds variables based on the goodness of fit determined via the adjusted R^2 values. The variable having the highest adjusted R^2 value was added first in the model, and the model development process stopped when adding new variables could not improve the adjusted R^2. Predictor variables are only included when the direction of the association is predetermined (e.g., positive for traffic load). The predictor variables in the LUR models were checked for p-value and collinearity. Here, p-value > 0.10 and variance inflation factor > 3 and Cook's D <1 was removed.

Text S3. Specific criteria for each selected object.

To capture dispersion characteristics of air pollutants, we included elements such as trees, grass, commercial buildings, and residential buildings. For example, dense tree coverage can create tree canyons that may trap air pollutants, while grassland areas suggest open spaces with potentially better dispersion. A high density of buildings can indicate the presence of street canyons causing accumulation of pollutants.

We included also potential emission-related objects: gas stations, factories, construction sites, shops. Chimneys reflect a potential emission source – residential combustion. Windows and streetlamps describe finer-scale building and street characteristics to help distinguish urban forms and functions. Water and ships are unique objects for Amsterdam, where canals connect the historic center and some ships may be powered by diesel engines, representing a unique emission source.

Traffic-related objects such as bikes, cars, trucks, and buses were selected to capture transportation activities. A higher presence of people and cyclists can imply lower car usage and thus lower emissions. Differentiating vehicle types (e.g., trucks vs. buses) allows refinement of emission factors. Traffic signs and traffic lights were included to represent vehicle status and behavior (e.g., idling or acceleration), which are not captured by traffic intensity data.

We acknowledge that there are likely many more objects that could provide insight into urban

air pollution. As our framework enables flexible, hypothesis-driven object selection, future studies may identify and test novel objects that reflect urban environmental factors beyond air pollution, such as noise and heat

Table S1: Spatial predictor variables with units, predefined directions of effect, and buffer sizes in Amsterdam.

Predictor variable	Abbreviation	Units	Direction of effect	Buffer	10th percentile	Mean	90th Percentile
Agricultural land area[1]	AGRI_	m^2	-	100	0	1159	0
				300	0	11288	0
				500	0	34779	64052
				1000	0	178942	649727
				5000	1710991	13290916	30287613
Airport area[1]	AIR_	m^2	+	5000	0	640794	1628347
Industry area[1]	INDUS_	m^2	+	100	0	2006	0
				300	0	17769	41347
				500	0	49074	180131
				1000	0	199831	730248
				5000	2902185	5182292	8091464
Natural and forested areas[1]	NATUR_	m^2	-	100	0	0	0
				300	0	0	0
				500	0	0	0
				1000	0	0	0
				5000	0	429332	2388024
Port area[1]	PORT_	m^2	+	100	0	2648	2516
				300	0	22447	85979
				500	0	59770	258949
				1000	0	226393	956817
				5000	0	6167718	13267782
Residential land area[1]	RES_	m^2	+	100	0	21152	31416
				300	0	183950	282742
				500	0	492795	785396
				1000	104805	1826998	3040804
				5000	17043122	32157817	46811645
Transportation area[1]	TRANS_	m^2	+	100	0	0	0
				300	0	0	0
				500	0	13457	2874
				1000	0	50061	233924
				5000	519010	1340506	2093995
Urban Green area[1]	URBG_	m^2	-	100	0	2351	7952
				300	0	25250	102385
				500	0	79234	268822
				1000	0	361378	852803
				5000	6248972	9362179	13883066
Water[1]	WATER_	m^2		100	0	0	0
				300	0	10238	30716
				500	0	37897	152537
				1000	0	222478	741893
				5000	2904140	8362718	12310634
Population density[2]	POP_	n	+	100	0	319	770
				300	0	2385	5355
				500	25	6091	13410
				1000	1485	21762	46115
				5000	146514	366674	580006
Traffic intensity on nearest road[3]	TRAFNEAR	Veh/day	+		137	9986	22487

Traffic intensity on nearest major road[3]	TRAFMAJOR	Veh/day	+		3115	14212	28042
Heavy-duty traffic intensity on nearest road[3]	HTRAFNEAR	Veh/day	+		0	619	1333
Heavy-duty traffic intensity on nearest major road[3]	HTRAFMAJOR	Veh/day	+		54	980	2138
Road length of all roads[3]	RDL_	m	+	25	39	66	100
				50	100	187	294
				100	254	636	966
				300	2029	4880	7083
				500	7893	13761	19090
				1000	29092	50344	68095
Road length of all major roads[3]	MRDL_	m	+	25	0	24	91
				50	0	69	198
				100	0	210	534
				300	0	1532	3554
				500	0	3787	8800
				1000	3015	13627	31882
Traffic intensity on all roads (sum of (traffic intensity * length of all segments)) [3]	TLOA_	(Veh/day)*m	+	25	0	634722	1579945
				50	6393	1815133	4797110
				100	78159	5494027	14290643
				300	3038453	36918277	1.01E+08
				500	13741938	44180744	87625101
				1000	62971674	166892417	324383228
Traffic intensity on all major roads (sum of (traffic intensity* length of all segments)) [3]	TMLOA_	(Veh/day)*m	+	25	0	543194	1387293
				50	0	1548534	4473085
				100	0	4550786	13415227
				300	0	12783795	26453420
				500	0	30877644	64537936
				1000	26948221	116664854	238567679
Heavy-duty traffic intensity on all roads (sum of (heavy-duty traffic intensity* length of all segments)) [3]	HLOA_	(Veh/day)*m	+	25	0	40889	59383
				50	0	115926	205867
				100	1607	336918	909792
				300	84212	2085219	8507687
				500	387896	2664654	6027990
				1000	2550331	10515869	25699398
Heavy-duty traffic intensity on major roads (sum of (heavy-duty traffic intensity*length of all segments) [3]	HMLOA_	(Veh/day)*m	+	25	0	35363	41295
				50	0	100488	141074
				100	0	286355	742416
				300	0	846066	1614863
				500	0	2033997	4769469
				1000	966747	8018542	22978460

[1]Source: CORINE (Copernicus Land Monitoring Service) 2018, raster 100m.

[2]Source: CBS (Central Bureau of Statistics Netherlands) 2017, 100m*100m.

[3]Source: NWB (National Road Network Netherlands) 2011, vehicles per day per segment (between intersections)

Table S2: Performance table of NO_2 models (μg/m^3) trained using mobile measurements and validated by 5-fold cross validation.

Temporal strategy	***algorithm***	***Features***	***5-fold Cross-validation***					
			R^2	*Change*	*MAE*	*Change*	*RMSE*	*Change*

SpecificY	*SLR*	*Classic*	*0.45*		*6.03*		*8.65*	
		Classic+visual	*0.47*	*0.02*	*5.93*	*1.7%*	*8.43*	*2.5%*
	RF	*classic*	*0.71*		*3.82*		*5.83*	
		Classic+visual	*0.72*	*0.01*	*3.80*	*0.5%*	***5.79***	*0.7%*
MostnearY	*SLR*	*classic*	*0.41*		*6.39*		*9.21*	
		Classic+visual	*0.43*	*0.02*	*6.32*	*1.1%*	*9.08*	*1.4%*
	RF	*classic*	*0.71*		*3.87*		*5.93*	
		Classic+visual	*0.71*	*0*	***3.78***	*2.3%*	*5.90*	*0.5%*
Season weighted	*SLR*	*classic*	*0.43*		*6.54*		*9.10*	
		Classic+visual	*0.46*	*0.03*	*6.43*	*1.7%*	*8.85*	*2.8%*
	RF	*classic*	*0.73*		*4.17*		*5.89*	
		Classic+visual	***0.75***	*0.02*	*4.15*	*0.5%*	*5.87*	*0.3%*

Table S3: Performance table of NO_2 models without traffic intensity features($\mu g/m^3$) trained using mobile measurements and validated by 5-fold cross validation.

Temporal strategy	***algorithm***	***Features***	***5-fold Cross-validation***					
			R^2	*Change*	*MAE*	*Change*	*RMSE*	*Change*
SpecificY	*SLR*	*Classic*	*0.33*		*6.69*		*9.49*	
		Classic+visual	*0.38*	*0.05*	*6.47*	*3.3%*	*9.11*	*4%*
	RF	*classic*	*0.68*		*4.23*		*6.56*	
		Classic+visual	*0.68*	*0*	*4.12*	*2.7%*	*6.54*	*0.3%*
MostnearY	*SLR*	*classic*	*0.32*		*6.92*		*9.93*	
		Classic+visual	*0.37*	*0.05*	*6.71*	*3.0%*	*9.57*	*3.6%*
	RF	*classic*	*0.68*		*4.25*		*6.68*	
		Classic+visual	*0.71*	*0.03*	*3.78*	*11.1%*	*5.87*	*12.1%*
Season weighted	*SLR*	*classic*	*0.31*		*7.29*		*10.03*	
		Classic+visual	*0.40*	*0.09*	*6.92*	*5.1%*	*9.36*	*6.7%*
	RF	*classic*	*0.73*		*4.36*		*6.11*	
		Classic+visual	*0.74*	*0.01*	*4.31*	*1.2%*	*6.03*	*1.3%*

Table S4. Performance of NO_2 models without traffic intensity features (µg/m³) validated by routine long-term measurements (Palmes).

Temporal strategy	***Algorithms***	***Features***	***All available Palmes***							***Palmes_33***							***SHAPLEY Ratio***
			# of Palmes	*R^2*	*Change*	*MAE*	*Change*	*RMSE*	*Change*	*# of Palmes*	*R^2*	*Change*	*MAE*	*Change*	*RMSE*	*Change*	
SpecificY	SLR	Classic	43	0.38		4.79		5.75		33	0.08		4.55		5.60		
		Classic+visual		0.42	0.04	4.67	2.5%	5.62	2.3%		0.08	0	4.38	3.7%	5.51	1.6%	
	RF	classic		0.60		5.26		7.10			0.56		5.83		7.28		
		Classic+visual		0.64	0.04	5.02	4.6%	6.92	2.5%		0.60	0.04	5.45	7.0%	6.90	5.2%	0.20
MostnearY	SLR	classic	65	0.35		4.68		5.89		33	0.08		4.57		5.55		
		Classic+visual		0.43	0.08	4.47	2.4%	5.48	7.0%		0.10	0.02	4.46	2.4%	5.60	-0.9%	
	RF	classic		0.56		5.83		8.04			0.55		5.60		7.12		
		Classic+visual		0.54	0.02	5.67	2.7%	7.90	1.8%		0.58	0.03	5.43	3.0%	6.93	2.7%	0.18
Season weighted	SLR	classic	38	0.15		4.98		5.93		33	0.10		4.62		5.43		
		Classic+visual		0.22	0.07	4.62	7.2%	5.51	7.1%		0.14	0.04	4.53	2.0%	5.42	0.2%	
	RF	classic		0.57		6.47		8.18			0.50		6.51		8.03		
		Classic+visual		0.59	0.02	6.02	7.0%	7.66	6.4%		0.51	0.01	6.24	4.2%	7.67	4.5%	0.21-0.23

Table S2: Coefficients of SLR with SpecificY street-view images for NO_2

Coefficients:

	Estimate	Std. Error	t value	Pr(>|t|)
(Intercept)	2.809e+00	6.360e-01	4.415	1.01e-05***
TMLOA_50	8.112e-07	3.839e-08	21.128	<2e-16***
POP_5000	2.089e-05	9.026e-07	23.149	<2e-16***
MRDL_25	3.743e-02	2.805e-03	13.342	<2e-16***
**wall.perc_pixels**	4.061e+01	1.828e+00	22.213	<2e-16***
TRAFNEAR	1.140e-04	6.907e-06	16.507	<2e-16***
PORT_500	4.818e-06	4.749e-07	10.146	<2e-16***
TLOA_1000	4.572e-09	3.521e-10	12.987	<2e-16***
**road.perc_pixels**	1.098e+01	8.616e-01	12.742	<2e-16***
MRDL_100	4.751e-03	4.719e-04	10.068	<2e-16***
INDUS_5000	4.006e-07	4.038e-08	9.923	<2e-16***
AGRI_5000	1.260e-07	1.245e-08	10.121	<2e-16***
**grass.perc_pixels**	1.238e+01	1.153e+00	10.738	<2e-16***
TRANS_100	1.616e-04	2.051e-05	7.878	3.51e-15***
TRAFMAJOR	3.602e-05	4.392e-06	8.203	2.48e-16***
URBG_500	-5.031e-06	5.397e-07	-9.322	<2e-16***
PORT_5000	1.650e-07	1.704e-08	9.679	<2e-16***
TRANS_5000	1.176e-06	1.209e-07	9.721	<2e-16***
**traffic.light**	1.874e+02	2.914e+01	6.431	1.30e-10***
**fence.perc_pixels**	1.807e+01	3.192e+00	5.662	1.52e-08***

Multiple R-squared: 0.4741, Adjusted R-squared: 0.4735

Signif. codes: 0 '***' 0.001 '**' 0.01 '*' 0.05 '.' 0.1 ' ' 1

Table S3: Coefficients of SLR under SpecificY Scenario without street-view images for NO_2

Coefficients:

	Estimate	Std. Error	t value	Pr(>|t|)
(Intercept)	7.945e+00	5.958e-01	13.333	<2e-16***
TMLOA_50	9.321e-07	3.768e-08	24.745	<2e-16***
POP_5000	1.847e-05	8.906e-07	20.733	<2e-16***
MRDL_25	4.775e-02	2.653e-03	18.005	<2e-16***
TRAFNEAR	1.461e-04	6.567e-06	22.254	<2e-16***
PORT_500	8.550e-06	4.559e-07	18.754	<2e-16***
TLOA_1000	4.630e-09	3.431e-10	13.495	<2e-16***
INDUS_5000	5.597e-07	3.905e-08	14.333	<2e-16***
MRDL_100	4.807e-03	4.798e-04	10.019	<2e-16***
AGRI_5000	1.184e-07	1.231e-08	9.624	<2e-16***
TRANS_100	1.849e-04	2.077e-05	8.905	<2e-16***

Multiple R-squared: 0.4473, Adjusted R-squared: 0.447

Signif. codes: 0 '***' 0.001 '**' 0.01 '*' 0.05 '.' 0.1 ' ' 1

Table S4: Coefficients of SLR under MostnearY Scenario with street-view images features for NO_2

Coefficients:

	Estimate	Std. Error	t value	Pr(>|t|)
(Intercept)	7.554e+00	3.484e-01	21.686	<2e-16***
TMLOA_50	7.362e-07	3.023e-08	24.359	<2e-16***
MRDL_100	5.813e-03	3.724e-04	15.604	<2e-16***
POP_5000	3.326e-06	1.380e-06	2.410	0.016*
TRAFNEAR	1.123e-04	5.664e-06	19.828	<2e-16***
**wall.perc_pixels**	3.559e+01	1.351e+00	26.329	<2e-16***
**road.perc_pixels**	1.159e+01	6.870e-01	16.869	<2e-16***
PORT_500	4.773e-06	3.444e-07	13.865	<2e-16***
TLOA_500	1.115e-08	8.043e-10	13.858	<2e-16***
MRDL_25	3.266e-02	2.318e-03	14.093	<2e-16***
TRANS_5000	1.616e-06	9.767e-08	16.549	<2e-16***
**grass.perc_pixels**	1.120e+01	9.481e-01	11.813	<2e-16***
PORT_5000	2.232e-07	1.680e-08	13.278	<2e-16***
**traffic.light**	1.722e-01	2.019e-02	8.531	<2e-16***
TRANS_100	1.552e-04	1.705e-05	9.099	<2e-16***
RES_5000	1.483e-07	1.842e-08	8.049	8.62e-16***
TRAFMAJOR	2.788e-05	3.683e-06	7.570	3.83e-14***
Multiple R-squared: 0.4533, Adjusted R-squared: 0.453				

Signif. codes: 0 ‘***’ 0.001 ‘**’ 0.01 ‘*’ 0.05 ‘.’ 0.1 ‘ ’ 1

Table S5: Coefficients of SLR under MostnearY Scenario without street-view images features for NO_2

Coefficients:

	Estimate	Std. Error	t value	Pr(>|t|)
(Intercept)	1.234e+01	2.388e-01	51.70	<2e-16***
TMLOA_50	8.300e-07	2.997e-08	27.70	<2e-16***
MRDL_100	6.136e-03	3.743e-04	16.40	<2e-16***
POP_5000	1.139e-05	3.662e-07	31.11	<2e-16***
TRAFNEAR	1.431e-04	5.300e-06	27.00	<2e-16***
PORT_500	5.973e-06	3.482e-07	17.15	<2e-16***
MRDL_25	4.491e-02	2.181e-03	20.59	<2e-16***
TLOA_500	1.439e-08	7.761e-10	18.55	<2e-16***
TRANS_5000	1.403e-06	9.325e-08	15.05	<2e-16***
PORT_5000	1.400e-07	1.305e-08	10.73	<2e-16***
INDUS_100	7.334e-05	7.227e-06	10.15	<2e-16***
Multiple R-squared: 0.4333, Adjusted R-squared: 0.4331				

Signif. codes: 0 ‘***’ 0.001 ‘**’ 0.01 ‘*’ 0.05 ‘.’ 0.1 ‘ ’ 1

Table S6: Coefficients of SLR during warm season with street-view images features for NO_2

Coefficients:

	Estimate	Std. Error	t value	Pr(>|t|)
(Intercept)	-4.606e+00	7.450e-01	-6.183	6.38e-10***

**TMLOA_50**	5.839e-07	4.036e-08	14.470	<2e-16***
**MRDL_50**	3.119e-02	9.943e-04	31.365	<2e-16***
POP_5000	2.839e-05	1.035e-06	27.430	<2e-16***
TRAFNEAR	1.637e-04	6.546e-06	25.006	<2e-16***
**wall.perc_pixels**	3.856e+01	1.847e+00	20.887	<2e-16***
MRDL_1000	1.895e-04	1.672e-05	11.332	<2e-16***
**road.perc_pixels**	1.055e+01	8.731e-01	12.080	<2e-16***
AGRI_5000	2.720e-07	1.423e-08	19.114	<2e-16***
PORT_5000	2.963e-07	1.762e-08	16.819	<2e-16***
INDUS_5000	4.937e-07	4.721e-08	10.459	<2e-16***
TMLOA_300	2.314e-08	2.297e-09	10.080	<2e-16***
AIR_5000	4.869e-07	4.869e-08	10.001	<2e-16***
TRANS_5000	9.838e-07	1.258e-07	7.823	5.36e-15***
**commercial.buildings**	1.957e-01	3.044e-02	6.430	1.30e-10***
Multiple R-squared: 0.3988, Adjusted R-squared: 0.3985				

Signif. codes: 0 ‘***’ 0.001 ‘**’ 0.01 ‘*’ 0.05 ‘.’ 0.1 ‘ ’ 1

Table S7: Coefficients of SLR during cold season with street-view images features for NO_2

Coefficients:

	Estimate	Std. Error	t value	Pr(>|t|)
(Intercept)	-1.246e+01	1.302e+00	-9.574	<2e-16***
TMLOA_25	2.209e-06	1.685e-07	13.106	<2e-16***
MRDL_100	8.233e-03	6.625e-04	12.425	<2e-16***
POP_5000	9.267e-06	2.897e-06	3.199	0.00138**
TRANS_1000	1.680e-05	1.050e-06	16.003	<2e-16***
**wall.perc_pixels**	3.429e+01	2.436e+00	14.071	<2e-16***
INDUS_5000	1.354e-06	8.244e-08	16.419	<2e-16***
**sky.perc_pixels**	7.843e+00	1.415e+00	5.546	2.99e-08***
WATER_1000	7.238e-06	4.585e-07	15.787	<2e-16***
TRAFMAJOR	4.839e-05	9.449e-06	5.121	3.09e-07***
AGRI_1000	4.627e-06	4.520e-07	10.236	<2e-16***
**road.perc_pixels**	1.222e+01	1.530e+00	7.988	1.50e-15***
MRDL_500	5.052e-04	7.851e-05	6.436	1.27e-10***
**tree.perc_pixels**	8.499e+00	1.537e+00	5.532	3.24e-08***
TRANS_5000	2.495e-06	2.519e-07	9.908	<2e-16***
RES_5000	2.132e-07	3.713e-08	5.741	9.64e-09***
NATUR_5000	-1.598e-06	2.337e-07	-6.840	8.33e-12***
RES_1000	2.183e-06	2.938e-07	7.431	1.16e-13***
**water.perc_pixels**	6.300e+01	1.350e+01	4.668	3.07e-06***
PORT_100	1.191e-04	2.017e-05	5.901	3.71e-09***
INDUS_100	9.323e-05	2.025e-05	4.606	4.15e-06***
TRAFNEAR	5.907e-05	1.362e-05	4.336	1.46e-05***
Multiple R-squared: 0.3283, Adjusted R-squared: 0.3271				

Signif. codes: 0 ‘***’ 0.001 ‘**’ 0.01 ‘*’ 0.05 ‘.’ 0.1 ‘ ’ 1

Table S8: Coefficients of SLR under SpecificY Scenario with street-view images for BC

Coefficients:

	Estimate	Std. Error	t value	Pr(>|t|)
(Intercept)	5.987e-01	2.197e-02	27.257	<2e-16***
TMLOA_50	4.969e-08	2.685e-09	18.509	<2e-16***
MRDL_25	2.413e-03	1.966e-04	12.277	<2e-16***
**wall.perc_pixels**	2.526e+00	1.272e-01	19.865	<2e-16***
TRAFNEAR	7.755e-06	4.520e-07	17.158	<2e-16***
**road.perc_pixels**	8.317e-01	6.055e-02	13.736	<2e-16***
MRDL_100	2.791e-04	3.296e-05	8.470	<2e-16***
INDUS_5000	2.584e-08	2.341e-09	11.038	<2e-16***
POP_5000	3.585e-07	3.071e-08	11.673	<2e-16***
**grass.perc_pixels**	7.430e-01	7.942e-02	9.355	<2e-16***
**traffic.light**	1.369e-02	1.590e-03	8.611	<2e-16***
TMLOA_500	3.887e-10	6.251e-11	6.217	5.16e-10***
**chimney**	2.501e-02	4.076e-03	6.136	8.60e-10***
**fence.perc_pixels**	1.181e+00	2.223e-01	5.315	1.08e-07***

Multiple R-squared: 0.3609, Adjusted R-squared: 0.3605

Signif. codes: 0 ‘***’ 0.001 ‘**’ 0.01 ‘*’ 0.05 ‘.’ 0.1 ‘ ’ 1

Table S9: Coefficients of SLR under SpecificY Scenario without street-view images for BC

Coefficients:

	Estimate	Std. Error	t value	Pr(>|t|)
(Intercept)	7.235e-01	2.194e-02	32.984	<2e-16***
TMLOA_50	5.493e-08	2.678e-09	20.511	<2e-16***
MRDL_25	3.357e-03	1.860e-04	18.047	<2e-16***
TRAFNEAR	8.565e-06	4.541e-07	18.861	<2e-16***
INDUS_5000	1.701e-08	2.628e-09	6.472	9.93e-11***
MRDL_100	2.992e-04	3.328e-05	8.992	<2e-16***
POP_5000	2.553e-07	3.056e-08	8.354	<2e-16***
INDUS_1000	7.974e-08	1.195e-08	6.673	2.58e-11***
TMLOA_500	4.253e-10	6.362e-11	6.685	2.36e-11***
TRANS_5000	6.337e-08	8.258e-09	7.674	1.75e-14***
PORT_5000	7.908e-09	1.116e-09	7.086	1.43e-12***

Multiple R-squared: 0.3437, Adjusted R-squared: 0.3434

Signif. codes: 0 ‘***’ 0.001 ‘**’ 0.01 ‘*’ 0.05 ‘.’ 0.1 ‘ ’ 1

Table S10: Coefficients of SLR under MostnearY Scenario with street-view images for BC

Coefficients:

	Estimate	Std. Error	t value	Pr(>|t|)
**(Intercept)**	-9.581e-02	5.001e-02	-1.916	0.0554.

TMLOA_50	4.861e-08	2.126e-09	22.862	<2e-16***
MRDL_25	2.559e-03	1.629e-04	15.707	<2e-16***
**wall.perc_pixels**	2.268e+00	9.730e-02	23.307	<2e-16***
TRAFNEAR	7.133e-06	3.711e-07	19.222	<2e-16***
MRDL_100	2.715e-04	2.606e-05	10.418	<2e-16***
**road.perc_pixels**	5.885e-01	5.056e-02	11.640	<2e-16***
PORT_5000	1.959e-08	1.079e-09	18.149	<2e-16***
**traffic.light**	1.327e-02	1.447e-03	9.170	<2e-16***
TMLOA_500	4.469e-10	5.411e-11	8.258	<2e-16***
TRANS_5000	8.148e-08	6.728e-09	12.111	<2e-16***
RES_5000	1.463e-08	8.099e-10	18.065	<2e-16***
**grass.perc_pixels**	6.536e-01	6.663e-02	9.809	<2e-16***
WATER_500	5.096e-07	4.409e-08	11.560	<2e-16***
AGRI_5000	1.038e-08	8.739e-10	11.874	<2e-16***
INDUS_5000	1.864e-08	2.247e-09	8.294	<2e-16***
**sky.perc_pixels**	2.271e-01	3.658e-02	6.209	5.41e-10***
Multiple R-squared: 0.3443, Adjusted R-squared: 0.3439				

Signif. codes: 0 ‘***’ 0.001 ‘**’ 0.01 ‘*’ 0.05 ‘.’ 0.1 ‘ ’ 1

Table S11: Coefficients of SLR under MostnearY Scenario without street-view images for BC

Coefficients:

	Estimate	Std. Error	t value	Pr(>|t|)
(Intercept)	3.205e-01	4.274e-02	7.498	6.66e-14***
TMLOA_50	4.998e-08	2.139e-09	23.372	<2e-16***
MRDL_25	3.596e-03	1.539e-04	23.374	<2e-16***
TRAFNEAR	7.841e-06	3.740e-07	20.968	<2e-16***
MRDL_100	3.104e-04	2.629e-05	11.806	<2e-16***
PORT_5000	2.013e-08	1.092e-09	18.433	<2e-16***
TRANS_5000	1.018e-07	6.603e-09	15.416	<2e-16***
TMLOA_500	5.496e-10	5.437e-11	10.108	<2e-16***
WATER_500	5.593e-07	4.200e-08	13.317	<2e-16***
RES_5000	9.948e-09	7.441e-10	13.369	<2e-16***
AGRI_5000	8.072e-09	8.282e-10	9.746	<2e-16***
Multiple R-squared: 0.3246, Adjusted R-squared: 0.3244				

Signif. codes: 0 ‘***’ 0.001 ‘**’ 0.01 ‘*’ 0.05 ‘.’ 0.1 ‘ ’ 1

Table S12: Coefficients of SLR during warm season with street-view images features for BC

Coefficients:

	Estimate	Std. Error	t value	Pr(>|t|)
(Intercept)	5.963e-01	1.850e-02	32.226	<2e-16***
TMLOA_50	5.147e-08	2.451e-09	20.995	<2e-16***
MRDL_25	2.878e-03	1.952e-04	14.745	<2e-16***
**wall.perc_pixels**	2.541e+00	1.235e-01	20.570	<2e-16***
TRAFNEAR	8.328e-06	4.361e-07	19.096	<2e-16***

**road.perc_pixels**	8.656e-01	5.953e-02	14.542	<2e-16***
PORT_5000	1.620e-08	1.034e-09	15.659	<2e-16***
MRDL_100	2.989e-04	3.222e-05	9.276	<2e-16***
TRANS_5000	7.572e-08	7.512e-09	10.079	<2e-16***
**traffic.light**	4.262e-02	4.522e-03	9.424	<2e-16***
**grass.perc_pixels**	6.780e-01	7.521e-02	9.015	<2e-16***
TLOA_500	5.202e-10	6.462e-11	8.050	8.60e-16***
WATER_300	7.884e-07	1.405e-07	5.612	2.03e-08***
Multiple R-squared: 0.3452, Adjusted R-squared: 0.3448				

Signif. codes: 0 '***' 0.001 '**' 0.01 '*' 0.05 '.' 0.1 ' ' 1

Table S13: Coefficients of SLR during cold season with street-view images features for BC

Coefficients:

	Estimate	Std. Error	t value	Pr(>|t|)
(Intercept)	-1.248e+00	1.166e-01	-10.703	<2e-16***
TMLOA_50	4.791e-08	4.277e-09	11.203	<2e-16***
INDUS_5000	1.118e-07	6.809e-09	16.425	<2e-16***
NATUR_5000	-7.724e-08	1.360e-08	-5.678	1.39e-08***
**road.perc_pixels**	9.355e-01	1.202e-01	7.783	7.69e-15***
**wall.perc_pixels**	2.944e+00	1.881e-01	15.649	<2e-16***
TRAFMAJOR	6.121e-06	6.392e-07	9.576	<2e-16***
MRDL_50	7.290e-04	1.337e-04	5.452	5.09e-08***
**chimney**	7.805e-02	9.181e-03	8.502	<2e-16***
TMLOA_1000	1.767e-10	5.285e-11	3.344	0.00083***
RES_5000	3.199e-08	2.346e-09	13.632	<2e-16***
**sky.perc_pixels**	1.124e+00	1.086e-01	10.349	<2e-16***
**tree.perc_pixels**	1.093e+00	1.241e-01	8.807	<2e-16***
INDUS_1000	1.215e-07	2.444e-08	4.973	6.69e-07***
WATER_1000	3.394e-07	3.345e-08	10.144	<2e-16***
AGRI_5000	1.888e-08	2.126e-09	8.880	<2e-16***
URBG_5000	-3.288e-08	4.288e-09	-7.670	1.86e-14***
**commercial.buildings**	2.236e-02	4.144e-03	5.396	6.95e-08***
TRANS_100	1.448e-05	2.515e-06	5.759	8.67e-09***
**grass.perc_pixels**	9.320e-01	1.958e-01	4.759	1.97e-06***
EEA_500	2.529e-05	5.472e-06	4.623	3.83e-06***
Multiple R-squared: 0.2226, Adjusted R-squared: 0.2212				

Signif. codes: 0 '***' 0.001 '**' 0.01 '*' 0.05 '.' 0.1 ' ' 1

Table S14: Coefficients of SLR under SpecificY Scenario with street-view images for UFP

Coefficients:

	Estimate	Std. Error	t value	Pr(>|t|)
(Intercept)	1.558e+03	7.303e+02	2.134	0.0329*
TMLOA_25	1.130e-03	1.977e-04	5.715	1.11e-08***
INDUS_5000	8.503e-04	6.056e-05	14.041	<2e-16***

HMLOA_100	1.889e-03	2.541e-04	7.434	1.10e-13***
TRAFMAJOR	4.402e-02	6.859e-03	6.418	1.42e-10***
**road.perc_pixels**	1.401e+04	1.391e+03	10.069	<2e-16***
RES_5000	1.778e-04	1.175e-05	15.131	<2e-16***
**grass.perc_pixels**	2.008e+04	1.955e+03	10.270	<2e-16***
TRANS_1000	7.787e-03	9.393e-04	8.290	<2e-16***
**chimney**	7.850e+02	1.002e+02	7.837	4.84e-15***
HMLOA_25	1.714e-02	2.526e-03	6.784	1.21e-11***
NATUR_1000	-1.445e-02	2.345e-03	-6.161	7.35e-10***
TRANS_5000	1.292e-03	1.986e-04	6.509	7.74e-11***
WATER_1000	1.887e-03	3.001e-04	6.287	3.30e-10***
**tree.perc_pixels**	4.586e+03	9.679e+02	4.738	2.18e-06***
AIR_5000	3.793e-04	8.145e-05	4.657	3.23e-06***

Multiple R-squared: 0.128, Adjusted R-squared: 0.1273

Signif. codes: 0 ‘***’ 0.001 ‘**’ 0.01 ‘*’ 0.05 ‘.’ 0.1 ‘ ’ 1

Table S15: Coefficients of SLR under SpecificY Scenario without street-view images for UFP

Coefficients:

	Estimate	Std. Error	t value	Pr(>|t|)
(Intercept)	9.022e+03	5.737e+02	15.727	<2e-16***
TMLOA_25	2.313e-03	1.292e-04	17.903	<2e-16***
INDUS_5000	8.826e-04	5.856e-05	15.071	<2e-16***
HMLOA_100	2.673e-03	2.284e-04	11.704	<2e-16***
TRAFMAJOR	4.574e-02	6.870e-03	6.658	2.85e-11***
RES_5000	1.038e-04	1.004e-05	10.334	<2e-16***
TRANS_1000	7.391e-03	9.200e-04	8.034	1.00e-15***
NATUR_1000	-1.470e-02	2.357e-03	-6.237	4.55e-10***
WATER_100	1.946e-01	3.527e-02	5.518	3.46e-08***
TRANS_5000	8.884e-04	1.939e-04	4.580	4.67e-06***

Multiple R-squared: 0.1146, Adjusted R-squared: 0.1142

Signif. codes: 0 ‘***’ 0.001 ‘**’ 0.01 ‘*’ 0.05 ‘.’ 0.1 ‘ ’ 1

Table S16: Coefficients of SLR under MostnearY Scenario with street-view images for UFP

Coefficients:

	Estimate	Std. Error	t value	Pr(>|t|)
(Intercept)	3.295e+02	6.432e+02	0.512	0.608
TMLOA_25	1.256e-03	1.663e-04	7.551	4.44e-14***
HMLOA_100	1.867e-03	2.108e-04	8.856	<2e-16***
INDUS_5000	8.342e-04	5.147e-05	16.208	<2e-16***
TRAFMAJOR	4.655e-02	5.588e-03	8.331	<2e-16***
**road.perc_pixels**	1.543e+04	1.090e+03	14.157	<2e-16***
RES_5000	1.939e-04	9.851e-06	19.685	<2e-16***
**grass.perc_pixels**	1.557e+04	1.667e+03	9.344	<2e-16***
TRANS_1000	8.692e-03	7.588e-04	11.456	<2e-16***

WATER_500	9.505e-03	9.652e-04	9.848	<2e-16***
**chimney**	5.611e+02	7.224e+01	7.767	8.26e-15***
**wall.perc_pixels**	1.767e+04	2.356e+03	7.501	6.53e-14***
HMLOA_25	1.432e-02	2.121e-03	6.752	1.49e-11***
AIR_5000	5.008e-04	5.992e-05	8.359	<2e-16***
TRANS_5000	1.282e-03	1.589e-04	8.065	7.61e-16***
AGRI_300	1.925e-02	2.523e-03	7.627	2.47e-14***
**tree.perc_pixels**	4.772e+03	7.919e+02	6.026	1.70e-09***
Multiple R-squared: 0.1348, Adjusted R-squared: 0.1343				

Signif. codes: 0 '***' 0.001 '**' 0.01 '*' 0.05 '.' 0.1 ' ' 1

Table S17: Coefficients of SLR under MostnearY Scenario without street-view images for UFP

Coefficients:

	Estimate	Std. Error	t value	Pr(>|t|)
(Intercept)	8.180e+03	4.554e+02	17.963	<2e-16***
TMLOA_25	2.439e-03	1.056e-04	23.088	<2e-16***
HMLOA_100	2.493e-03	1.882e-04	13.248	<2e-16***
INDUS_5000	8.911e-04	5.009e-05	17.791	<2e-16***
TRAFMAJOR	4.804e-02	5.586e-03	8.601	<2e-16***
INDUS_300	1.197e-02	1.549e-03	7.726	1.14e-14***
RES_5000	1.268e-04	8.502e-06	14.917	<2e-16***
TRANS_1000	8.473e-03	7.652e-04	11.073	<2e-16***
WATER_500	9.126e-03	9.485e-04	9.622	<2e-16***
AGRI_100	1.659e-01	2.067e-02	8.024	1.06e-15***
AIR_5000	4.192e-04	5.834e-05	7.184	6.91e-13***
Multiple R-squared: 0.1244, Adjusted R-squared: 0.1241				

Signif. codes: 0 '***' 0.001 '**' 0.01 '*' 0.05 '.' 0.1 ' ' 1

Table S18: Coefficients of SLR during warm season with street-view images features for UFP

Coefficients:

	Estimate	Std. Error	t value	Pr(>|t|)
(Intercept)	1.412e+03	7.556e+02	1.869	0.061690.
TMLOA_25	1.591e-03	1.728e-04	9.210	<2e-16***
TRANS_5000	2.765e-03	1.984e-04	13.933	<2e-16***
HTRAFMAJOR	5.382e-01	8.179e-02	6.580	4.79e-11***
INDUS_5000	1.125e-03	6.455e-05	17.436	<2e-16***
NATUR_1000	-1.832e-02	2.146e-03	-8.536	<2e-16***
WATER_500	3.821e-03	2.136e-03	1.788	0.073733.
HMLOA_100	1.939e-03	2.399e-04	8.083	6.58e-16***
TRAFNEAR	9.268e-02	1.288e-02	7.198	6.24e-13***
POP_1000	1.606e-01	1.065e-02	15.080	<2e-16***
AGRI_5000	1.705e-04	1.406e-05	12.128	<2e-16***
AIR_5000	6.723e-04	7.343e-05	9.155	<2e-16***

WATER_1000	4.456e-03	5.684e-04	7.840	4.68e-15***
TMLOA_1000	4.646e-06	6.237e-07	7.449	9.66e-14***
**grass.perc_pixels**	1.626e+08	4.424e+07	3.676	0.000237***
Multiple R-squared: 0.09929,	Adjusted R-squared: 0.09885			

Signif. codes: 0 ‘***’ 0.001 ‘**’ 0.01 ‘*’ 0.05 ‘.’ 0.1 ‘ ’ 1

Table S19: Coefficients of SLR during cold season with street-view images features for UFP

Coefficients:

	Estimate	Std. Error	t value	Pr(>|t|)
(Intercept)	-1.122e+04	1.897e+03	-5.916	3.38e-09***
TMLOA_25	1.576e-03	2.560e-04	6.155	7.73e-10***
RES_5000	4.369e-04	3.264e-05	13.387	<2e-16***
HMLOA_500	1.343e-04	3.323e-05	4.040	5.37e-05***
INDUS_1000	1.932e-03	4.671e-04	4.136	3.56e-05***
INDUS_5000	9.748e-04	1.200e-04	8.123	4.95e-16***
NATUR_5000	-1.805e-03	2.584e-04	-6.985	3.00e-12***
AGRI_5000	1.400e-04	3.299e-05	4.245	2.20e-05***
**wall.perc_pixels**	3.140e+04	4.047e+03	7.757	9.34e-15***
**sky.perc_pixels**	1.266e+04	2.107e+03	6.011	1.90e-09***
**tree.perc_pixels**	1.385e+04	2.303e+03	6.013	1.87e-09***
HMLOA_50	6.924e-03	1.179e-03	5.872	4.42e-09***
**road.perc_pixels**	8.700e+03	2.306e+03	3.773	0.000162***
Multiple R-squared: 0.1058, Adjusted R-squared: 0.105				

Signif. codes: 0 ‘***’ 0.001 ‘**’ 0.01 ‘*’ 0.05 ‘.’ 0.1 ‘ ’ 1

Table S20. Correlation table of traffic intensity features against selected visual features.

	BIKE	CAR	TREE	RESIDENTIAL.BUILDINGS	WINDOW	CAR.PERC_PIXELS	SKY.PERC_PIXELS	BUILDING.PERC_PIXELS	ROAD.PERC_PIXELS	SIDEWALK.PERC_PIXELS	GRASS.PERC_PIXELS	BICYCLE.PERC_PIXELS
**HHOLD_100**	0.52	0.42	0.42	0.59	0.52	0.32	-0.48	0.64	-0.2	0.33	-0.38	0.45
**HHOLD_300**	0.53	0.4	0.42	0.56	0.48	0.31	-0.48	0.6	-0.17	0.33	-0.4	0.46
**HHOLD_500**	0.52	0.38	0.41	0.54	0.46	0.29	-0.48	0.58	-0.15	0.32	-0.4	0.46
**HHOLD_1000**	0.53	0.35	0.39	0.52	0.45	0.26	-0.48	0.57	-0.13	0.31	-0.41	0.47
**TLOA_25**	-0.08	-0.11	-0.21	-0.14	-0.16	-0.13	0.19	-0.22	0.37	-0.22	-0.07	-0.07
**HLOA_25**	-0.08	-0.09	-0.2	-0.11	-0.11	-0.09	0.19	-0.18	0.26	-0.19	-0.04	-0.07
**TMLOA_25**	-0.12	-0.13	-0.25	-0.17	-0.18	-0.14	0.22	-0.25	0.38	-0.25	-0.05	-0.11
**HMLOA_25**	-0.09	-0.09	-0.2	-0.12	-0.11	-0.09	0.19	-0.19	0.26	-0.2	-0.04	-0.08
**TLOA_50**	-0.09	-0.12	-0.23	-0.15	-0.16	-0.14	0.18	-0.22	0.35	-0.21	-0.07	-0.08
**HLOA_50**	-0.09	-0.11	-0.22	-0.13	-0.13	-0.1	0.18	-0.2	0.27	-0.2	-0.03	-0.08
**TMLOA_50**	-0.12	-0.14	-0.26	-0.18	-0.18	-0.15	0.2	-0.26	0.36	-0.24	-0.04	-0.11
**HMLOA_50**	-0.1	-0.11	-0.22	-0.13	-0.13	-0.1	0.18	-0.21	0.27	-0.21	-0.03	-0.09
**TLOA_100**	-0.06	-0.11	-0.22	-0.12	-0.12	-0.11	0.13	-0.19	0.29	-0.19	-0.04	-0.06
**HLOA_100**	-0.1	-0.13	-0.24	-0.14	-0.13	-0.11	0.16	-0.21	0.24	-0.21	0.03	-0.08
**TMLOA_100**	-0.12	-0.14	-0.27	-0.18	-0.17	-0.13	0.17	-0.24	0.31	-0.22	-0.01	-0.11
**HMLOA_100**	-0.11	-0.13	-0.24	-0.15	-0.14	-0.11	0.16	-0.23	0.24	-0.21	0.03	-0.1
**TLOA_300**	-0.02	-0.06	-0.15	-0.07	-0.06	-0.05	0.05	-0.07	0.2	-0.1	-0.07	-0.01
**HLOA_300**	-0.12	-0.12	-0.23	-0.16	-0.14	-0.09	0.15	-0.18	0.2	-0.18	0.05	-0.1
**TMLOA_300**	-0.12	-0.11	-0.22	-0.16	-0.15	-0.09	0.13	-0.17	0.23	-0.15	-0.01	-0.1
**HMLOA_300**	-0.15	-0.13	-0.24	-0.18	-0.16	-0.09	0.17	-0.21	0.2	-0.19	0.06	-0.12
**TLOA_500**	0	-0.04	-0.1	-0.04	-0.04	-0.03	0	-0.01	0.15	-0.05	-0.09	0.01
**HLOA_500**	-0.14	-0.13	-0.22	-0.18	-0.16	-0.09	0.15	-0.18	0.17	-0.16	0.08	-0.11
**TMLOA_500**	-0.12	-0.1	-0.19	-0.17	-0.15	-0.08	0.11	-0.15	0.19	-0.11	0	-0.1

**HMLOA_500**	-0.17	-0.13	-0.24	-0.21	-0.18	-0.1	0.17	-0.22	0.18	-0.17	0.09	-0.14
**TLOA_1000**	0.09	0.03	-0.02	0.04	0.03	0.03	-0.12	0.09	0.13	0	-0.14	0.08
**HLOA_1000**	-0.15	-0.12	-0.22	-0.19	-0.16	-0.09	0.13	-0.19	0.18	-0.17	0.1	-0.12
**TMLOA_1000**	-0.09	-0.06	-0.14	-0.13	-0.12	-0.04	0.03	-0.11	0.17	-0.08	-0.02	-0.07
**HMLOA_1000**	-0.2	-0.13	-0.24	-0.23	-0.2	-0.09	0.16	-0.24	0.18	-0.18	0.12	-0.16
**TRAFNEAR**	-0.07	-0.1	-0.21	-0.13	-0.15	-0.11	0.18	-0.2	0.32	-0.21	-0.05	-0.07
**HTRAFNEAR**	-0.08	-0.08	-0.19	-0.11	-0.11	-0.08	0.19	-0.17	0.23	-0.18	-0.01	-0.07
**DINVNEAR**	0	0.01	0.01	-0.01	-0.01	0.01	0	0	0	0.01	-0.01	0.01
**TRAFMAJOR**	-0.07	-0.07	-0.18	-0.11	-0.1	-0.06	0.12	-0.15	0.15	-0.15	0	-0.06
**HTRAFMAJOR**	-0.1	-0.1	-0.2	-0.12	-0.11	-0.08	0.17	-0.18	0.12	-0.17	0.06	-0.08
**DINVMAJOR**	0.02	-0.01	0	-0.01	0	-0.01	-0.01	0.01	0	0.01	-0.01	0.03

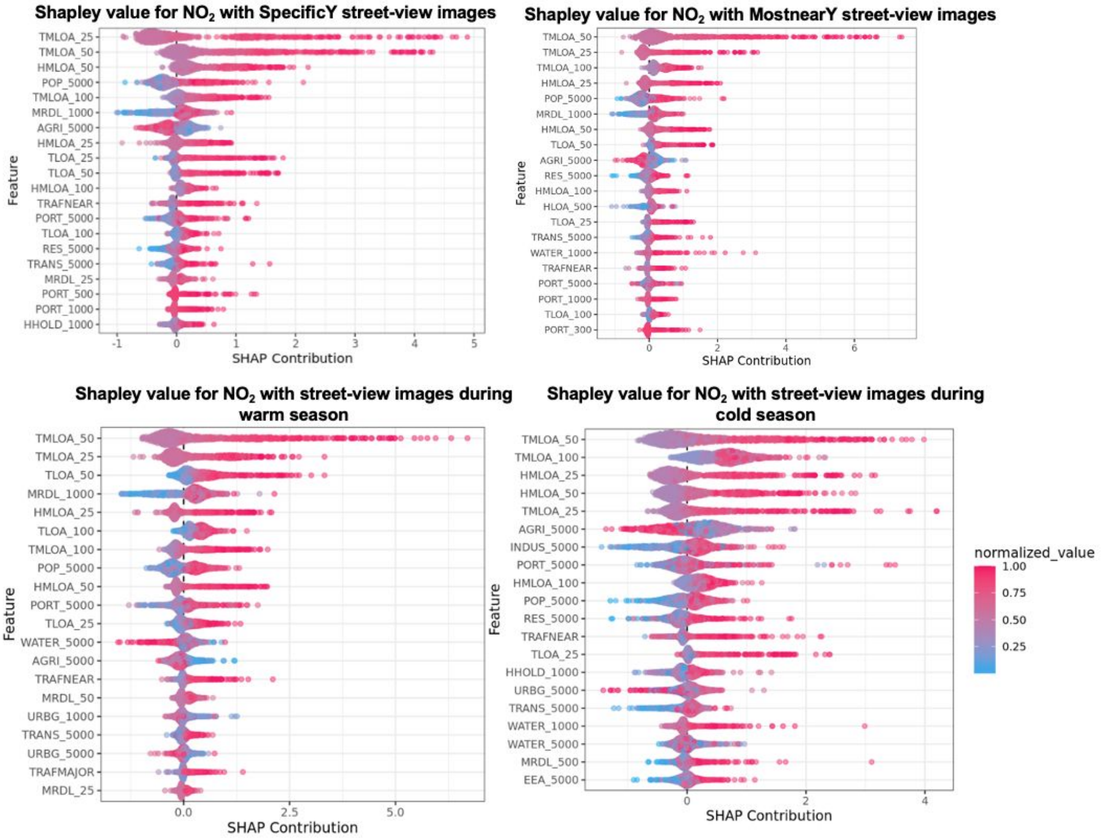

Figure S1: Global Shapley summary plot of RF with SpecificY for NO_2

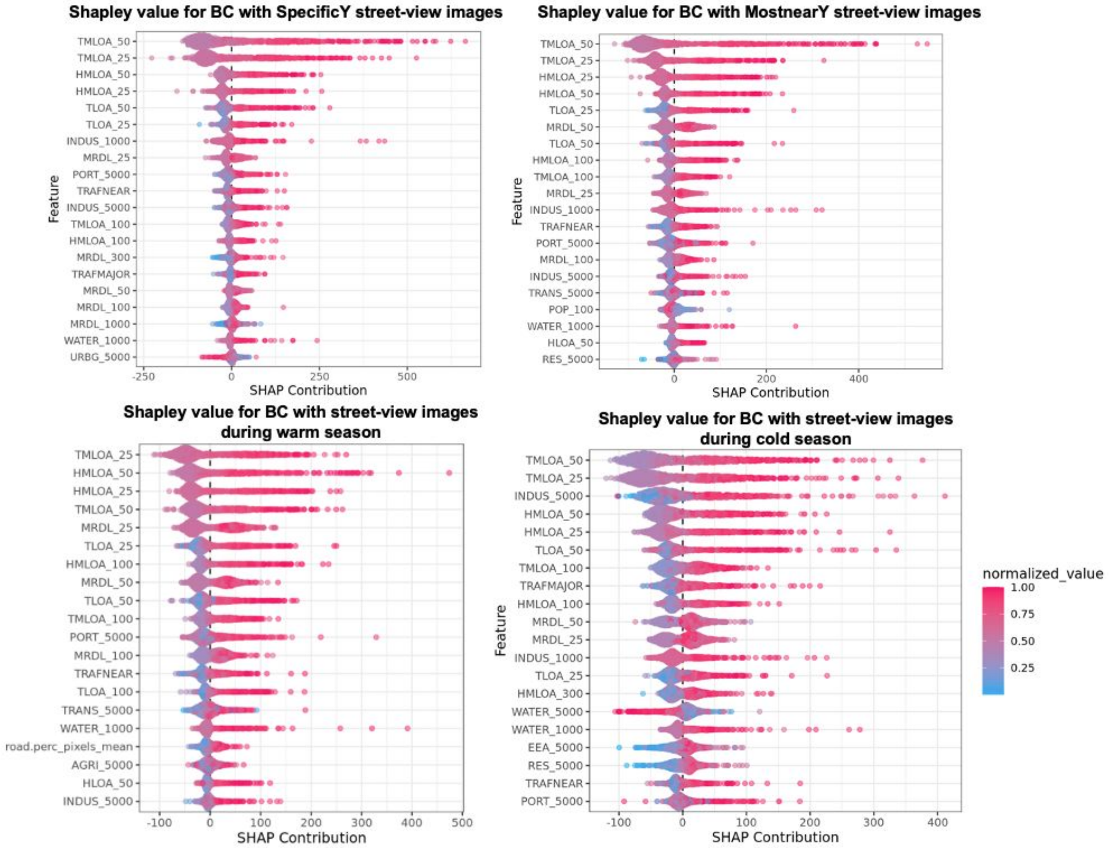

Figure S2: Global Shapley summary plot of RF with street-view images for BC.

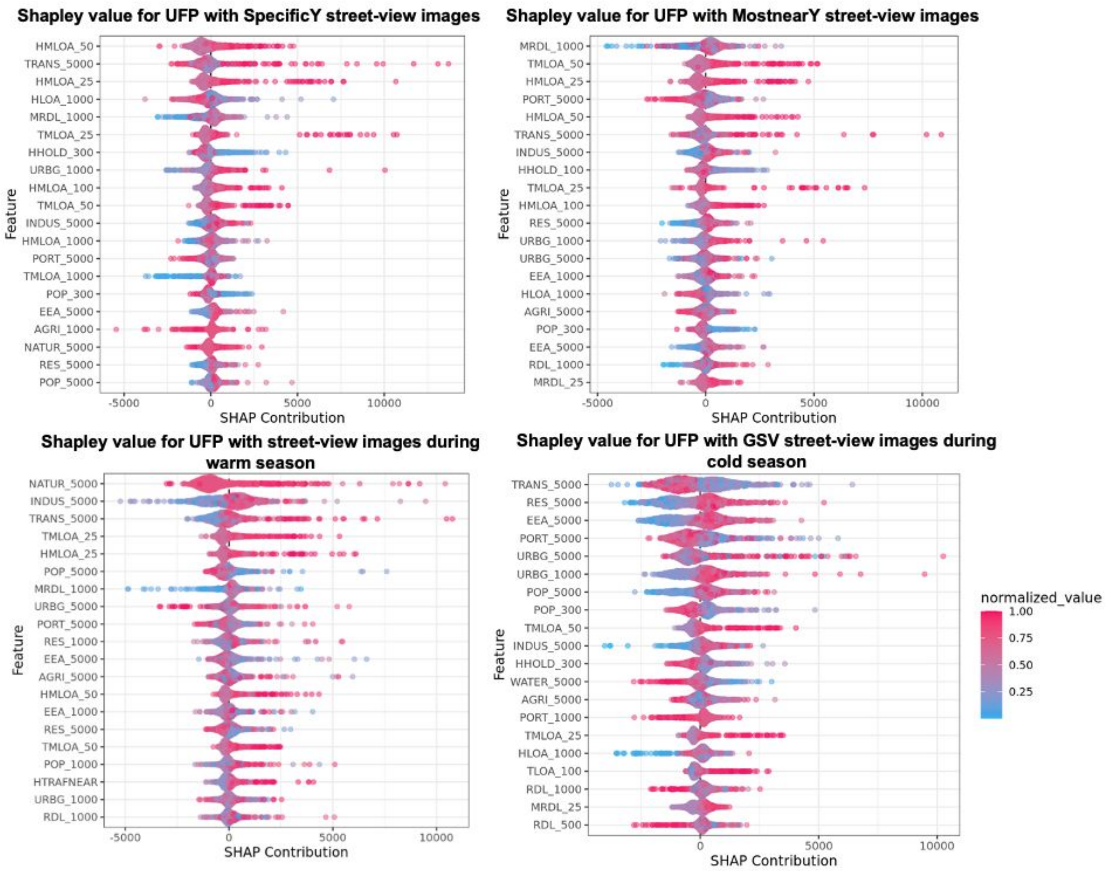

Figure S3: Global Shapley summary plot of RF with street-view images for UFP.
